# Health in Yemen: losing ground in war time

**DOI:** 10.1186/s12992-018-0354-9

**Published:** 2018-04-25

**Authors:** Charbel El Bcheraoui, Aisha O. Jumaan, Michael L. Collison, Farah Daoud, Ali H. Mokdad

**Affiliations:** Institute for Health Metrics and Evaluation, University of Washington, 2301 5th Ave, Seattle, WA 98121 USA

**Keywords:** War, Conflict, Maternal and child health, Malnutrition, Vaccine coverage, Mortality

## Abstract

**Background:**

The effect of the ongoing war in Yemen on maternal and child health (MCH) has not been comprehensively assessed. Providing a situational analysis at the governorate level is critical to assist in planning a response and allocating resources.

**Methods:**

We used multiple national- and governorate-level data sources to provide estimates of 12 relevant MCH indicators in 2016 around child vaccination, and child and maternal nutritional status, and the change in these estimates for the period 2013–2016 based on shock variables including change in gross domestic product, burden of airstrikes per 1000 population, change in access to untreated water sources and unimproved toilets, and change in wheat flour prices. We also used findings from the Global Burden of Disease 2016 study.

**Results:**

Vaccine coverage decreased for all antigens between 2013 and 2016 among children 12–23 months. The largest decrease, 36·4% for first-dose measles vaccine, was in Aden. Among children under the age of five, incidence of diarrhea was at 7·0 (5·5–8·9) episodes per person-year. The prevalence of moderate and severe child anemia ranged from 50·9% (24·9–73·1) in Sana’a City to 97·8% (94·1–99·2) in Shabwah in 2016. Prevalence of underweight among women of reproductive age ranged from 15·3% (8·1–24·6) in Sana’a city to 32·1% (24·1–39·7) in Hajjah, with a national average of 24·6% (18·7–31·5).

**Conclusions:**

The war and siege on Yemen has had a devastating impact on the health of women and children. Urgent efforts to secure food, essential medicines, antibiotics, deworming medicine, and hygiene kits, and cold chains for immunization are needed. Yemen is in dire need of clean water and proper sanitation to reduce the spread of disease, especially diarrhea.

**Electronic supplementary material:**

The online version of this article (10.1186/s12992-018-0354-9) contains supplementary material, which is available to authorized users.

## Background

Yemen is one of the poorest countries in the Middle East. It ranked as the 160th country (of 188) based on the Human Development Index in 2014 [1]. It is estimated that 35% of its population was living below the national poverty line in 2015 [2] and almost half of the population lacks access to sufficient and nutritious food [3]. Yemen is divided into 21 governorates – Abyan, Aden, Al-Baidha, Aldhalae, Al-Hodeida, Al-Jawf, Al-Mahrah, Al-Mahwit, Amran, Dhamar, Hadramout, Hajjah, Ibb, Lahj, Mareb, Reimah, Sadah, Sana’a, Sana’a City, Shabwah, and Taiz – with their own local health departments that report to the central Ministry of Public Health and Population (MOPHP).

Yemen has experienced multiple conflicts that intensified around 2010 [4], with large protests taking place in 2011, internal fighting in 2012–2014, and a war and siege that started in 2015 and continue to date [5]. The war and siege have had a devastating impact on every vital sector in Yemen including agriculture, service, and industry, which faced large-scale destruction and significant cost increases [6, 7]. Social service delivery and the already-weak health system have been deeply disrupted. Long power outages affected provision of most services, with a direct impact on the cold chain and the operations of clinics and hospitals. In parallel, water supply, sanitation, irrigation, and agricultural services were all hindered, leading to the deterioration of population health, especially women’s and children’s health [8, 9].

The last nationwide health survey was a Demographic Health Survey (DHS) conducted in 2013 [10]. The Global Burden of Disease (GBD) 2016 study estimated that the under-5 mortality rate (U5MR) rose from 48.9 deaths per 1000 in 2013 to 53·4 in 2015, a rate previously observed in 2009 [11]. While GBD provides some insight at the situation of maternal and U5MR, it is limited to the national level, and does not estimate most maternal and child health (MCH) indicators. Other studies have shown that the prevalence of children who were underweight increased from 39 to 45%, while wasting increased from 16·3 to 20·4% between 2013 and 2016; the percentage of children under 5 who had recent diarrhea increased from 31·2 to 42·7%, and the percentage of children aged 12–23 months who were fully vaccinated declined from 38·2 to 22·4% for the same period [12, 13].

Current estimates of MCH are of limited quality and geography making it difficult to measure the effect of war, taking evidence-based decisions, and allocating resources appropriately. For instance, Nutrition Status and Mortality Surveys were conducted only in two governorates in 2015, five in 2016, and only one in 2017. Hence, we used multiple data sources to provide estimates of several MCH indicators in 2016, and the change of these estimates from 2013 when DHS was conducted. Our analysis provides a comprehensive assessment of the health situation at the governorate level that is critical for planning and prioritizing programs to respond to health needs and properly allocate limited resources.

## Methods

We focused our analyses on maternal and child health to estimate the following indicators: For children, we focused on 1) vaccine coverage, including third-dose polio vaccine (Polio 3); third-dose diphtheria, tetanus, and pertussis (DTP3); third-dose pneumococcal vaccine; and first-dose measles vaccines among children 12–23 months old, 2) global acute malnutrition (GAM) stunting among children under 5 years old, 3) anemia among children 6–59 months old, and 4) under-5 mortality (U5MR). Specifically for measles vaccine, data was available for first-dose only. For women of childbearing age (15–49 years old), we focused on 1) underweight as measured by body mass index (BMI) < 18·5 kg/m^2^, 2), moderate malnutrition as measured by middle upper arm circumference 21–22·9 cm, 3) acute malnutrition as measured by middle upper arm circumference < 21 cm, and 4) maternal mortality.

### Data sources

We used all available national and governorate-level data sources. We also obtained numerous datasets, reports, and surveillance data from the MOPHP. Additional file 1: Annex 1 details all data reviewed and their use in this report. Data sources included administrative data, epidemiological surveillance reports, number of casualties reported by different organizations, health surveys conducted by different groups in Yemen, counts of internally displaced people (IDP), and economic indicators such as gross domestic product (GDP), change in wheat flour prices, and food insecurity.

We used 2013 as our baseline since it was before the major onset of unrest, war, and siege and because we have a nationally and governorate-representative Demographic and Health Survey (DHS). Yearly estimates for 2014–2016 were then predicted based on a set of covariates and using all collected data during this period. We used shock variables in our models to account for their impact on health. The shock variables included 1) change in populations based on internally displaced persons (IDP) for 2015 and 2016; 2) change in GDP for 2014–2016; 3) change in wheat flour prices, 4) changes in severe food insecurity for 2013–2016, 5) change in access to untreated water sources and unimproved toilets, and 6) number of airstrikes per 1000 population. Number of airstrikes, and change in wealth and in access to untreated water sources and unimproved toilets are presented in Additional file 2: Annex 2.

### Statistical analysis

We used ensemble models in all our analyses using three different modeling strategies based on data availability. For example, for vaccine coverage and nutritional indicators, we used a backward elimination beta regression ensemble model including SDI split indices and relevant covariates. We then picked the best-fitting model for each estimate based on the coefficient of multiple determination or R-squared. For vaccine coverage, the best-performing model included U5MR, administrative vaccine data, and a correction factor based on administrative vaccine data. For child nutrition, the final model included diarrhea, U5MR, and maternal anemia. For women’s nutritional indicators, the final model included maternal anemia and underweight (BMI < 18·5 kg/m^2^).

For some variables, we used a crosswalk from the Global Burden of Disease (GBD) 2016 study national findings for Yemen to generate estimates for each governorate. GBD 2015 introduced a Socio-demographic Index (SDI) for each country, and we created a similar index for each governorate for the crosswalk [14]. The Governorates’ SDI levels were a summary index based on maternal education, fertility, and wealth indices from the 2013 DHS.

The model was: Y_2013 + t_ = β_0, 2013_ + β_i, 2013_X_i, 2013 + t_ + β_j, 2013_X_j, 2013 + t_ + ε, where Y is the predicted outcome variable for year *2013 + t* (*t* varies from 0 to 3). The model contained four components: an intercept β_0, 2013_, fixed covariates effects β_i, 2013_, and β_j, 2013_, and a residual ε. The covariates were socio-demographic indices – maternal education, wealth, and fertility – and biological variables such as U5MR.

We computed 95% confidence intervals for all estimates by bootstrapping 1000 samples of each model. Analyses were performed using R Studio, and maps were created through R and ArcGIS. We present findings by governorates in our report and provide maps of change for each indicator from 2013 to 2016.

Statistical codes used to generate estimates can be available upon request. Detailed methodology is provided in Additional file 3: Annex 3.

## Results

Yearly estimates, 95% confidence intervals, and percent change between 2013 and 2016 for all indicators are presented in Additional file 4: Annex 4.

### Child health

#### Vaccine coverage, children 12–23 months

**Fig. 1:**
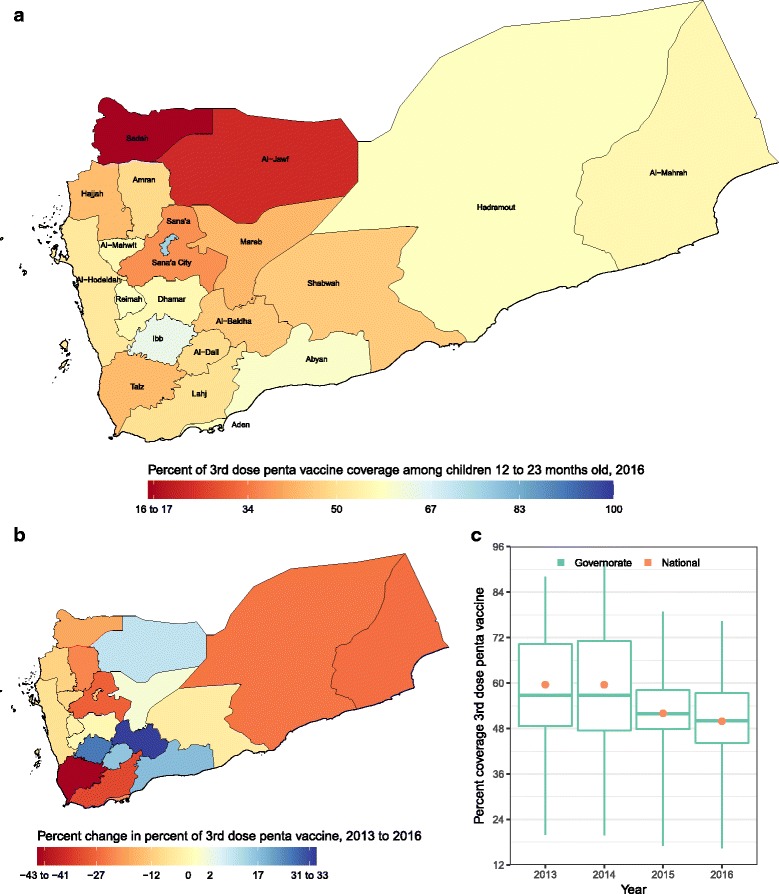
**a** DTP3 coverage in 2016; (**b**) percent change in DTP3 coverage from 2013 to 2016; (**c**) DTP3 coverage in 2013, 2014, 2015, and 2016. In panel (**c**), the boxes indicate the 25th, 50th, and 75th percentile across all governorates while the lines indicate the full range across governorates and the dots indicate the national-level coverage

At the national level, vaccine coverage decreased for all antigens between 2013 and 2016 in Yemen. Disparities were observed both between antigens and between governorates. The highest vaccine coverage for any antigen was in Sana’a City for DTP3 (Fig. 1), estimated at 76·3% (59·7–86·6), followed by third-dose polio vaccine at 73·0% (63·9–80·6) in 2016. The lowest vaccine coverage for any antigen was in Sadah for the third dose of pneumococcal vaccine, at 14·0% (6·7–22·0).

The largest estimated decrease, 42·6% (75·7% in 2013 to 43·4% in 2016), was in Taiz for DTP3 vaccine. The largest estimated increase, 212·7% (8·9% in 2013 to 27·8% in 2016), was in Al-Jawf for third-dose pneumococcal vaccine. As in Sadah, Mareb, Ibb, Al-Baidha, Al-Dali, and Lahj, the increase in coverage for some of the antigens was due either to small populations or to very low coverage in 2013. Coverage of third-dose pneumococcal vaccine in Sadah increased from 12·7% (5·4–20·0) in 2013 to 14·0% (6·7–22·0) in 2016. However, coverage decreased for all antigens in Sana’a City. Estimates for first-dose measles vaccine, third-dose polio vaccine, and third-dose pneumococcal vaccine are presented in Additional file 5: Annex 5, Figures S1, S2 and S3.

#### Diarrheal diseases, children under 5 years

**Fig. 2:**
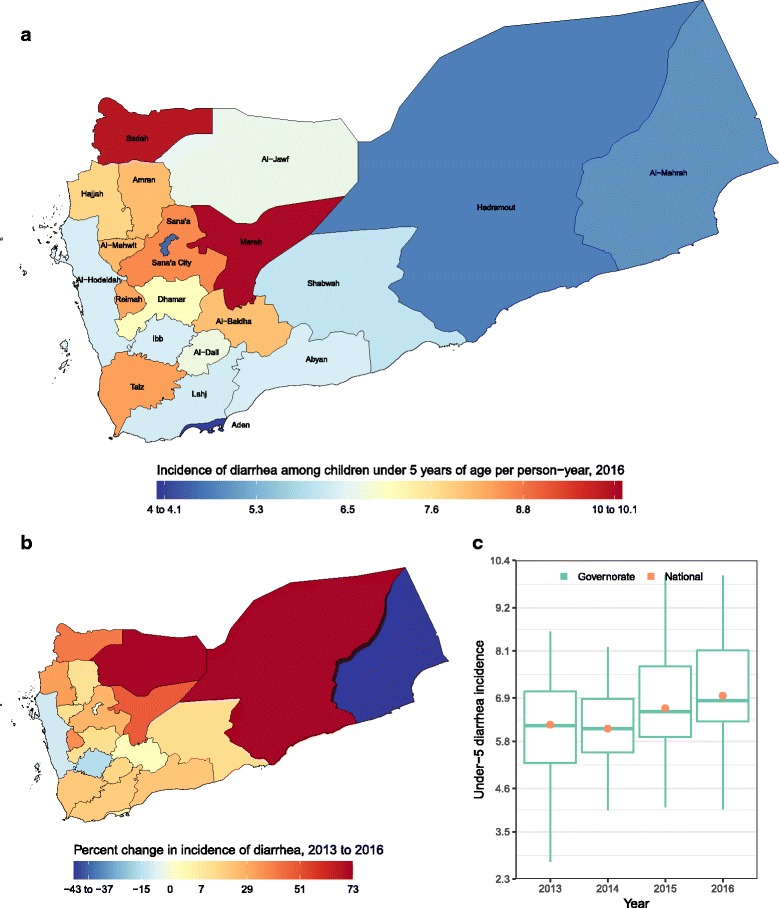
**a** Incidence of diarrhea episode per person-year in 2016; (**b**) percent change in incidence of diarrhea episode per person-year from 2013 to 2016; **c** Incidence of diarrhea episode per person-year in 2013, 2014, 2015, and 2016. In panel (**c**), the boxes indicate the 25th, 50th, and 75th percentile across all governorates while the lines indicate the full range across governorates and the dots indicate the national-level coverage

Incidence of diarrhea ranged from 4·1 (1·8–7·0) to 10·0 (6·2–13·4) episodes per person-year, with a national average of 7·0 (5·5–8·9), in 2016 (Fig. 2), and 11.9% increase since 2013. Mareb and Aden had the highest and lowest incidences: 10·0 (6·2–13·4) and 4·1 (1·8–7·0), respectively. The largest increase and decrease in incidence were estimated for Hadramout and Al-Mahrah at 73·2 and − 42·6%, respectively.

#### Global acute malnutrition (GAM) – stunting, children under 5 years

Prevalence of GAM stunting ranged from 33·1% (20·6–45·9) to 64·8% (58·7–70·6) in 2016. The national average increased from 46·5% (45·1–48·0) to 52·3% (44·0–58·5), a 12·5% increase from 2013 (Additional file 5: Annex 5, Figure S4). SAM stunting increased in 20 of the 22 governorates, with Al-Mahrah witnessing the largest increase at 160% (23·1% in 2013 to 38·6% in 2016), and Reimah with the highest prevalence of 64·8% (58·7–70·6) in 2016.

#### Moderate and severe anemia, children under 5 years

Moderate and severe anemia among children under 5 years of age increased in 17 of the 22 governorates between 2013 and 2016, with the largest increase observed in Sana’a at 36·1% (68·4% in 2013 to 93·1% in 2016). At the national level, an increase of 30.3% was estimated between 2013 and 2016. The prevalence of moderate and severe anemia ranged from 50·9% (24·9–73·1) to 97·8% (94·1–99·2), with governorates closest to the coast carrying the heaviest burden in 2016 (Additional file 5: Annex 5, Figure S5).

#### Under-5 mortality

**Fig. 3:**
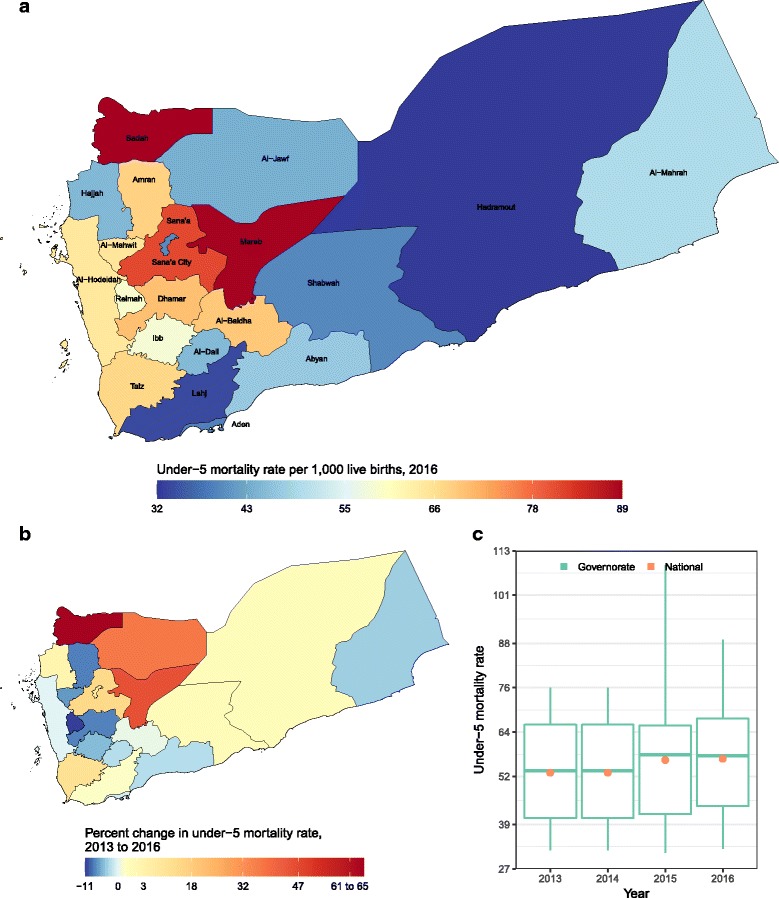
**a** Rate of under-5 mortality per 100,000 under-5 population in 2016; (**b**) percent change in rate of under-5 mortality from 2013 to 2016; **c** Rate of under-5 mortality in 2013, 2014, 2015, and 2016. In panel (**c**), the boxes indicate the 25th, 50th, and 75th percentile across all governorates while the lines indicate the full range across governorates and the dots indicate the national-level coverage

Child mortality increased nationally from 53 deaths per 1000 live births in 2013 to 56·8 in 2016, ranging from 32·4 in Hadramout to 88·9 in Sadah (Fig. 3). Overall, it increased in 10 of the 22 governorates.

### Women’s health, women 15–49 years old

#### Underweight – BMI < 18·5 kg/m^2^

**Fig. 4:**
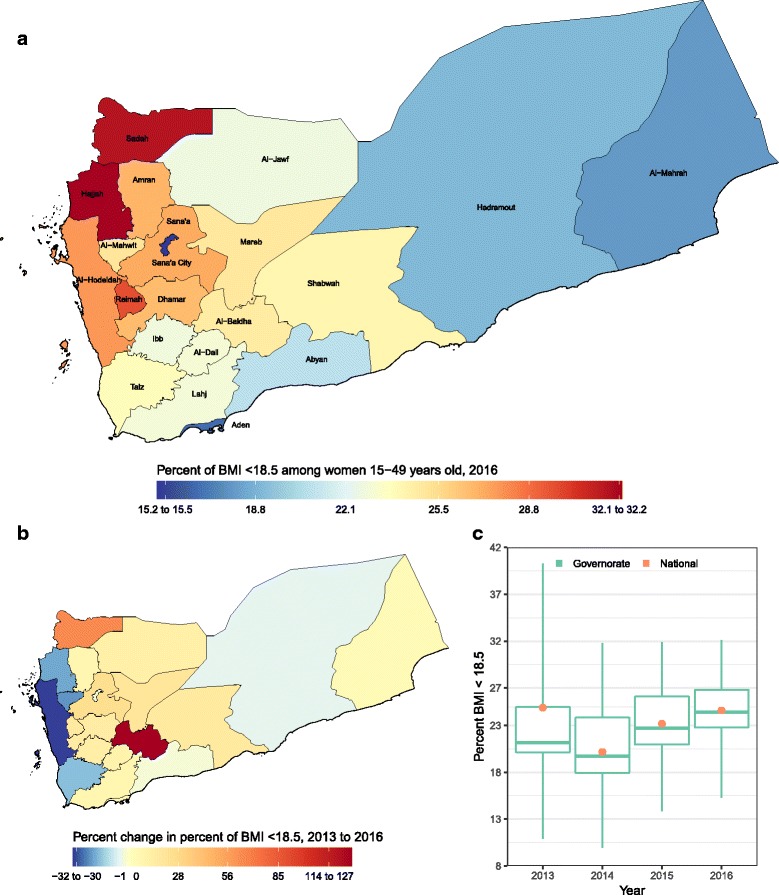
**a** Prevalence of maternal underweight – BMI < 18.5 kg/m^2^ in 2016; (**b**) percent change in underweight from 2013 to 2016; **c** Prevalence of underweight in 2013, 2014, 2015, and 2016. In panel **c**, the boxes indicate the 25th, 50th, and 75th percentile across all governorates, while the lines indicate the full range across governorates and the dots indicate national-level coverage

Prevalence of underweight ranged from 15·3% (8·1–24·6) to 32·1% (24·1–39·7) in 2016, with a national average of 24·6% (18·7–31·5), and the highest prevalence in Hajjah in 2016 (Fig. 4). Prevalence of underweight decreased by 1.2% at the national level, but increased in 14 of the 22 governorates, with the largest increase observed in Al-Baidha.

#### Moderate malnutrition – middle upper arm circumference 21·0–22·9 cm

Prevalence of moderate malnutrition ranged from 16·0 (15·3–16·7) to 31·8 (30·8–34·4) in 2016, with a national average of 23·7 (23·2–24·7), and the highest prevalence in Hajjah in 2016 (Additional file 5: Annex 5, Figure S6). Prevalence of moderate malnutrition increased by 16.3% between 2013 and 2016 at the national level, and in 16 of the 22 governorates, with the largest increase observed in Al-Baidha.

#### Severe malnutrition – middle upper arm circumference < 21·0 cm

Prevalence of severe malnutrition ranged from 5·8 (3·7–7·1) to 18·0 (13·9–28·3) in 2016, with a national average of 11·4 (9·7–14·0), and the highest prevalence in Hajjah in 2016 (Additional file 5: Annex 5, Figure S7). Prevalence of severe malnutrition decreased by 1.6% at the national level, but increased in 10 of the 22 governorates, with the largest increase observed in Al-Baidha.

#### Maternal mortality

**Fig. 5:**
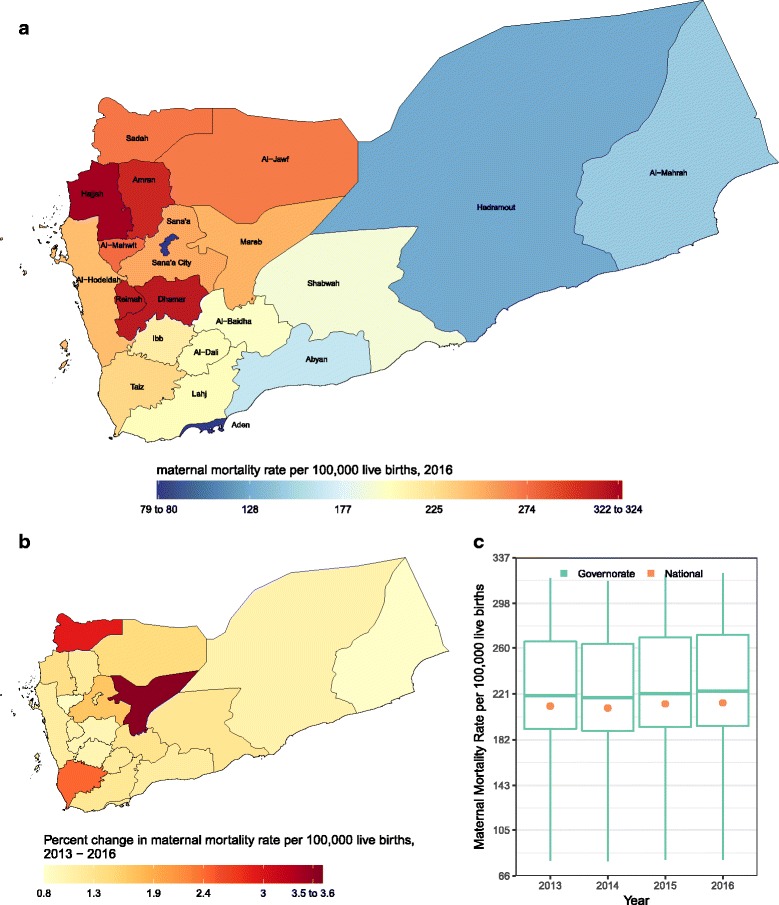
**a** Rate of maternal mortality per 100,000 live births in 2016; **b** percent change in rate of maternal mortality from 2013 to 2016; **c** Rate of maternal mortality in 2013, 2014, 2015, and 2016. In panel (**c**), the boxes indicate the 25th, 50th, and 75th percentile across all governorates while the lines indicate the full range across governorates and the dots indicate the national-level coverage

Maternal mortality increased in all Yemeni governorates, ranging from 79·9 deaths per 100,000 live births in Sana’a City to 323·9 in Hajjah, with a national average of 213·4 deaths per 100,000 live births in 2016, a 1.3% increase from 2013 (Fig. 5).

## Discussion

Our analysis is the first to provide a comprehensive assessment of the impact of war and siege on Yemen at the national and governorate levels. Indeed, this war and siege have had a devastating impact on child and maternal health. The decreasing vaccination rates among children reflect how basic public health activities have been impeded, while the increasing diarrheal disease incidence among children, and worsening nutrition status among mothers and children reflect the effect of war on the infrastructure as well as food availability and access. Hence, our findings call for stabilizing Yemen by first stopping the war and ending the siege. Moreover, urgent efforts are needed to provide protein-rich food to the affected population, provide access to clean water and sanitation, increase immunization activities, and ensure that essential medicines, antibiotics, deworming medicine, and hygiene kits are available to those who need them.

Between 2011 and 2012, with the political unrest, Al-Qaeda in the Arabian Peninsula took control of multiple governorates, especially in southern and central provinces, including Abyan and Al-Baidha, and fighting occurred in multiple Governorates in the north, including Sadah, Al Jawf, and Hajjah [15]. This led to a disruption of already weak health services, including the immunization program. A measles outbreak with over 4300 cases and 155 deaths occurred between January 2011 and March 2012 with high mortality rates [16, 17]. To respond to the outbreak, Yemen launched a successful national measles immunization campaign in mid-March, reaching 94% of the 8·2 million targeted children aged 6 months to 10 years [18]. Therefore, several 2013 indicators were very low due to unrest, and the increases observed in some indicators in 2016 may reflect this fact. Indeed, several activities that usually target maternal and child health were the most affected in these governorates during 2011–2012.

The decrease in GDP since 2013 and the shortage of food, fuel, and essential goods due to the siege have led to increases in market prices. Several rapid needs’ assessments have indicated a severe state of food insecurity for most Yemeni households [19–21]. Our estimates of nutrition status among women and children reflect this situation well. In Yemen, families undergoing economic hardships tend to send family members to rural areas to stay with extended family where the cost of living is cheaper and access to health care is limited. However, this strains the host families’ resources and worsens the situation further.

The decrease in vaccination rates is not surprising. The health system in Yemen has suffered greatly, and different sources estimate that about 55% of health facilities are currently not fully functional [22–24]. This is compounded by the fact that only about 50% of the population in Yemen had access to health care before the escalation in violence that started in March 2015. Furthermore, many non-governmental organizations working in the health sector have been hit as well. By January 2017, four Doctors Without Borders hospitals had been hit by airstrikes, resulting in casualties including deaths, injuries, and ultimately evacuation of medical staff [25]. However, despite the destruction of 25 to 55% of health facilities in the country, and more than 3·1 million internally displaced people, the MOPHP implemented a number of National and subnational immunization days and deployed mobile clinics in 2015 and 2016 [26, 27]. During 2015, the country estimates that 30 to 35% of coverage was ensured through five rounds of outreach activities [22]. It is important to keep in mind that even with these tremendous efforts, the cold chain may have been affected. Therefore, more such campaigns are needed and verification of effective coverage should be used (i.e., dried blood spots) to ensure that children are protected [28].

Our estimates of increased incidence in diarrheal diseases are a direct result of the war on Yemen’s infrastructure. This situation deteriorated even more in 2017 with the massive cholera outbreak that started in April. As of August 6, 2017, 468,638 suspected cholera cases and 1944 deaths had been reported [29, 30]. This outbreak started in October 2016 but did not reach alarming levels, and was thought to have tapered off by March 2017 before it spiked to a much higher level by the end of April. prior to the crisis, access to treated water sources and improved toilets was limited to 50% of households. With a rate of airstrikes up to 29.1 per 1000 population, destruction of infrastructure cannot be avoided. Hence, controlling this increase in diarrheal diseases and the continued cholera outbreak which started in 2016 will require tremendous efforts from all stakeholders, including the World Health Organization, the MOPHP, and others, and will require financial support from donors.

It is important to mention that our estimates of child mortality are not in line with those reported by us in our GBD 2016 study. According to GBD 2016, child mortality decreased over the 2013–2016 period in Yemen. GBD uses strong and rigorous statistical modeling to provide its estimates. However, this is an unfolding situation and an ongoing war. GBD has a yearly deadline for including new data to meet publication timeline. This analysis was conducted later and included more datasets than those used in GBD 2016 for Yemen. These new data sources and others will be included in GBD 2017.

### Limitations

Our study has some limitations. First, some of the data we used are of poor quality and limited geographical coverage. For example, we had access to many surveillance reports for diarrheal diseases, but some were incomplete or did not cover the whole governorate. Secondly, some of the data sources contradicted each other and we had to drop some outliers. Thirdly, we used the number of deaths due to conflict provided by the International Institute for Strategic Studies – 16,598. We suspect that, while this number is still higher than the one reported by WHO, it is still a large underestimation of the actual number of deaths because there are no death registries and estimates of deaths from health facilities only capture those who die in health facilities, covering only about 25% of the population [31, 32]. It has been reported that around 75 people are either killed or injured in the conflict every day [33]. Furthermore, in 2016, Oxfam reported that 10,000 additional children under 5 died due to preventable diseases, and UNICEF estimated that a child in Yemen dies every 10 min. During 2015 to August 2016, 19,958 airstrikes on Yemen were reported. It has also been reported that airstrikes have hit more civilian than non-civilian sites [34]. Finally, some of our estimates are based on statistical modeling due to lack of data and poor quality of some. However, this study used extensive sources of data and applied a rigorous methodology.

### Recommendations

Our findings point to several recommendations. Firstly, there is an urgent need to stop the war and restore peace in Yemen. Secondly, to improve health and reduce the burden, the blockade on imports of food, fuel, medicine, and essential goods should be lifted, and Hodeida port’s capacity to receive the needed supplies should be improved. Finally, Yemen is in dire need of financial support and donation to relief activities to improve the health situation and help with rebuilding the country and its infrastructure. One of the most immediate recommendations is to lift the embargo on working with the MOPH in Sana’a. Currently the MOPH section based in Sana’a controls most activities in the largely populated areas and has trained health promoters and social workers. These assets should be used and supported to prevent and control diseases.

There are also several recommendations that would improve the health situation in Yemen. Here we present them as short-, medium-, and long-term.

In the short term, stakeholders need to increase delivery of protein-rich food sources to reduce malnutrition and anemia and their long-term effects; increase the availability and distribution of clean water to control diarrheal diseases; support the immunization program to improve vaccine coverage by strengthening the cold chain, transport capacity, manpower, and providing vaccines and financial support to administer them; and secure essential medicines, antibiotics, deworming medicine, and hygiene kits. In this period, the surveillance systems should include measures for effective coverage such as dried blood spots to ensure that vaccines have a proper cold chain. This phase should include rapid health assessment surveys to identify areas of need and set the stage for all later activities.

In the medium term, stakeholders should ensure protection of children as many have lost their parents or caregivers, improve access to clean water and sanitation, establish new surveillance systems linked to laboratories to detect outbreaks and signs of danger, improve the health information systems, and rebuild health facilities. In this phase, there is a need for comprehensive national surveys such as DHS, multiple indicator cluster surveys, and WHO STEPwise approach to surveillance, and these should cover the whole spectrum of morbidity and mortality. Special attention should be given to mental health as its burden will be amplified due to the war. In parallel, there needs to be an assessment of the effect of the war on the health system to map functional health facilities and allocate resources accordingly.

In the longer run, there is a need for a Marshall plan to improve health and security in Yemen. Such a plan needs to examine the impact on farming, factories, animal health to sustain meat and milk production, roads, bridges, and environmental impact. Yemen needs to restore, strengthen, and expand its health system to meet the primary, secondary, and tertiary health care needs of its population. The country should also have sustainable access to treated water and access to improved toilets. Economically, it will be important to protect local farmers from price drops due to food distribution. Prices of commodities might drop if the international community is successful in securing food donations. This will have a devastating impact on agriculture in Yemen. The worst-case situation is that some farmers may shift to growing Qat as it is likely to be more profitable in the long run. Therefore, the UN agencies should buy local food at a higher price to ensure that food production increases in Yemen. This intention should be widely advertised so farmers will plant their fields and be followed through using a system to purchase the crops.

## Conclusions

The war and blockade on Yemen have had devastating impact on MCH. All MCH indicators have fallen below pre-crisis levels. Of 252 governorate-indicators presented in this study, 188 (74.6%) have deteriorated. Vaccination rates have decreased, and diarrheal diseases incidence has increased among children. Anemia and malnutrition have increased among mothers and children. Both maternal and U5MR have increased. Protein-rich food is urgently needed for the affected population. Children in Yemen need protection from infectious diseases. Immunization activities need to be secured and provided to ensure disease control and prevent potential outbreaks. Besides vaccines, and with the destruction that affected the health system in Yemen, essential medicines, antibiotics, deworming medicine, and hygiene kits should be available to those who need them. Urgent efforts to secure these products and public health and health care services are urgently needed. Furthermore, Yemen needs support to provide clean water and proper sanitation to reduce the burden of disease. The international community should urgently secure sustainable food access to all Yemenis and target households with internally displaced people. Tremendous efforts are needed from all stakeholders, including the World Health Organization, the MOPHP, and others, and financial support is required from donors to reverse this situation.

## Additional files

Additional file 1: **Table S1.** Data sources and their use in “Health in Yemen: losing ground in war time”. (DOCX 17 kb)

Additional file 2: **Figure S1.** Proportion of total airstrikes in [A] 2015 and [B] 2016, and airstrikes per 1000 population in [C] 2015 and [D] 2016 in Yemen, **Figure S2.** Change in population due to internally displaced persons from [A] 2013–2015 and [B] 2015–2016 in Yemen. **Figure S3.** Percent change in [A] severe food insecurity, [B] wheat flour price, [C] wealth index, 2013–2016 in Yemen. **Figure S4.** Percent change in access to [A] untreated water sources based on SDI, [B] unimproved toilets based on SDI, 2013–2016 in Yemen. (DOCX 1629 kb)

Additional file 3: Health in Yemen: losing ground in war time, detailed methodology. (DOCX 25 kb)

Additional file 4: Estimates of maternal and child health indicators and their 95% confidence intervals, 2013–2016, and percent change from 2013 to 2016 by governorate, Yemen. (DOCX 80 kb)

Additional file 5: [A] Maps of maternal and child health indicators' estimates in 2016; [B] percent change from 2013 and 2016; [C] estimates in 2013, 2014, 2015, and 2016. In panel [C], the boxes indicate the 25th, 50th, and 75th percentile across all governorates, while the lines indicate the full range across governorates and the dots indicate national-level estimates. (DOCX 1389 kb)
