# Supplementary material for: Health in Yemen: losing ground in war time
Source: Global Health. 2018 Apr 25;14:42. doi: 10.1186/s12992-018-0354-9 (PMC5918919; doi:10.1186/s12992-018-0354-9)
Supplement: Supplementary file 1 — Table S1. Data sources and their use in “Health in Yemen: losing ground in war time”. (DOCX 17 kb) [file 12992_2018_354_MOESM1_ESM.docx]

Table S1: Data sources and their use in “Health in Yemen: losing ground in war time”

| **Data source** | **Type of data** | **Topic** | **Year** | **Geography** | **Usage** | **Reason for not using** |
| --- | --- | --- | --- | --- | --- | --- |
| DHS 2013 | Survey | Immunization, SDI, mortality, nutritional status | 2013 | National, Governorate | Basis for estimates modeling, all models |  |
| Weekly Epidemiological Bulletin (eDEWS) | Administrative data | ID surveillance, cholera (2016) | 2013 - 2017 | National, Governorate | Diarrhea calculation (not used in final) | Not enough data |
| MoPHP Immunization Coverage | Administrative data | Immunization | 2011 - 2016 | National, Governorate | Immunization model input |  |
| Integrated Surveillance | Administrative data | ID surveillance | 2013 - 2016 | National, Governorate | Diarrhea calculation (not used in final) | Not enough data |
| Nutrition Status and Mortality Surveys | Survey | nutritional status, mortality | 2012 - 2016 | 2013 - Dhamar, Mhweit 2014 - Hajjah, Hodeidah 2015 - Al-Baidha, Hodeidah 2016 - Aldhalae, Hodeidah, Sadah, Sana'a, Taiz 2017 - shabwah |  | Not enough data |
| WHO snapshot of health facilities based reported deaths and injuries | Report | Health facility reported child mortality numbers due to war | 2015 - 2016 | National, Governorate | additional child deaths added to child mortality calculation (not used in final) | Replaced by GBD child deaths |
| Ministry of Planning and International Cooperation Socio-economic update | Report | Change in GDP, food security | 2016 | National | wealth index calculation |  |
| Task Force on Population Movement | Report | Change in population due to displacements | 2015-2017 | National, Governorate | wealth index calculation, child mortality calculation |  |
| Vulnerability and Needs Assessment | Survey | Nutritional Status/ Food | 2016 | Lahj, Taiz |  | Not enough data |
| Annual Statistical Health Report | Report | Mortality, nutritional status - ID surveillance - | 2013-2014 | National National, Governate | Diarrhea calculation (not used in final) | Not enough data |
| Comprehensive Food Security Survey (CFSS) | Survey | Nutritional Status, food | 2012, 2014 | National, Governorate |  | Used Famine Early Warning Systems Network Food Security outlook instead |
| Yemen Nutrition Cluster | Collection of Surveys | Nutritional Status | 2015 | National, Governorate |  | Collection of surveys with no additional data |
| WHO and UNICEF estimates of immunization coverage | Estimates | Immunization | 2005-2015 | National |  | Not applicable |
| WHO Weekly Cholera Situation Reports | Report | Cholera | 2016-2017 | Abyan, Aden, Al-Baidha, Aldhalae, Sana'a City, Hodeidah, Amran, Hajjah, Ibb, Lahj, Sana'a, Taiz, Ibb, dhamar, al-jawf |  | Did not model cholera |
| Food and Agriculture Organization (FAO) Yemen Situation Report | Report | Agriculture/Food | 2016-2017 | National |  | Not applicable |
| Famine Early Warning Systems Network Yemen Food Security Outlook | Report | Agriculture/Food | 2016-2017 | National, Governorate | maternal anemia calculation |  |
| WFP Yemen Market Watch Report | Report | Agriculture/Food | 2016-2017 | Governorate | wealth index calculation |  |
| Yemen Emergency Food Security and Nutrition Assessment (EFSNA) | Survey | Agriculture/Food, Nutrition | 2016 | National, Governorate | maternal anemia calculation |  |
| FAO Integrated Food Security Phase Classification | Report | Agriculture, Nutrition | 2015-2016 | National |  | Not applicable |
| WHO estimates of NTDs | Estimates | NTDs | 2015 | National |  | Did not model NTDs |
| Global Trachoma Mapping Project | Administrative data | Trachoma | 2015 | National, Governorate |  | Did not model trachoma |
| Malaria Dataset (MoPHP?) | Administrative data | Malaria | 2011-2015 | National, Governorate |  | Did not model malaria |
| ACTED Rapid Needs Assessment | Survey | Food | 2015 | Al Jawf |  | Not enough data |
| DHAid Needs Assessment | Survey | WASH / Food | 2016 | Ibb |  | Not enough data |
| INTERSOS Protection Needs Assessment | Survey | WASH / Food | 2015 | Taiz |  | Not enough data |
| IRC Rapid Needs Assessment | Survey | WASH / Food | 2015 | Shabwah |  | Not enough data |
| Rapid Multi-Cluster Assessment | Survey | WASH / Food | 2015 | Lahj |  | Not enough data |
| Islamic Help Multi-Cluster Needs Assessment | Survey | WASH / Food | 2015 | Taiz |  | Not enough data |
| Save the Children Multi-Sector Rapid Needs Assessment | Survey | WASH / Food | 2015 | Amran |  | Not enough data |
| UNHCR Rapid Multi-Cluster Assessment | Survey | WASH / Food | 2015 | Ibb |  | Not enough data |
| Relief International Rapid Constraints Analysis | Survey | Health System, Nutrition, WASH, Food | 2016 | Sana'a, Amran, Hajjah |  | Not enough data |
| Yemen Foundation for Development and Response (YFDR) Rapid Needs Assessment | Survey | WASH / Food | 2016 | Sana'a City |  | Not enough data |
| YFCA Rapid Needs Assessment | Survey | Health System | 2016 | Hajjah, Hodeidah |  | Not enough data |
| Life Makers Meeting Place (LMMPO) Multi Indicator Rapid Assessment | Survey | WASH / Food | 2016 | Taiz |  | Not enough data |
| Oxfam Rapid WASH Assessment Report | Survey | WASH / Food | 2015 | Taiz |  | Not enough data |
| WHO Yemen Daily Situation Report on Emergency Health Response | Report | Mortality | 2015 | National, Governorate |  | Data summarized in snapshots |
| OCHA Humanitarian Bulletin | Report | Food | 2015-2017 | National |  | Only national level data |
| OCHA Shipping Report | Report | Food | 2015 | National |  | Only national level data |
| Cases of AWD/Cholera | Administrative data | Cholera | 2016-2017 | Sana'a City, Taiz, Hodeidah, Aden, Lahj, Al-Baidha, Sana'a, Hajjah, Ibb, Amran, Aldhalae, Dhamar, Reimah, Abyan, Al-Jawf |  | Did not model cholera |
| MoH, WHO, UNICEF Cholera Outbreak Situation Report | Report | Cholera | 2017 | Amran, Hajjah, Al-Mhweit, Sana'a, Sana'a City, Hodeidah, Reimah, Dhamar, Ibb, Al-Baidha, Abyan, Lahj, Taiz, Aden, Ibb, Al-Jawf |  | Did not model cholera |
| WFP Yemen Food Security Situation | Report | Food | 2015 | National, Governorate |  | Not applicable |
| WFP Bulletin | Report | Food | 2015 | National, Governorate |  | Not applicable |
| OCHA Governorate Dashboard | Report | Health Systems | 2017 | National, Governorate |  | Did not use health system data |
| Leishmaniasis Cases | Administrative data | NTDs | 2013-2015 | National, Governorate |  | Did not model NTDs |
| Food Security Technical Secretariat (FSTS) Monthly Market Monitoring Bulletin | Report | Food | 2016 | Sana'a City, Hodeidah, Hajjah, Dhamar, Hadramout, Lahj, Taiz |  | Not enough data |
| OCHA Situation Report | Report | WASH / Food | 2015 | National |  | Only national level data |
| WHO EMRO Leishmaniasis Cases | Administrative data | NTDs | 2014-2015 | National |  | Did not model NTDs |
| WHO HeRAMS | Survey | Health Systems | 2016 | Abyan, Aden, Al-Baidha, Aldhalae, Sana'a City, Hodeidah, Amran, Hajjah, Ibb, Lahj, Sana'a, Taiz, Ibb, Mareb, al-jawf, Sada'a |  | Did not use health system data |
| FSA, WFP, OCHA food security status | Report | Food | 2014 | National, Governorate | maternal anemia calculation |  |
| Yemen Data Project airstrike dataset | Administrative data | NA | 2015-2016 | Governorate | wealth index calculation, untreated water and toilet calculation |  |
| NAFSA guide to education systems | Report | NA | 2010 | National | education index calculation |  |
| MoPHP supplementary immunization campaigns | Administrative data | Immunization | 2006 - 2016 | National, Governorate | report |  |
