# Supplementary material for: Health in Yemen: losing ground in war time
Source: Global Health. 2018 Apr 25;14:42. doi: 10.1186/s12992-018-0354-9 (PMC5918919; doi:10.1186/s12992-018-0354-9)
Supplement: Supplementary file 2 — Figure S1. Proportion of total airstrikes in [A] 2015 and [B] 2016, and airstrikes per 1000 population in [C] 2015 and [D] 2016 in Yemen, Figure S2. Change in population due to internally displaced persons from [A] 2013–2015 and [B] 2015–2016 in Yemen. Figure S3. Percent change in [A] severe food insecurity, [B] wheat flour price, [C] wealth index, 2013–2016 in Yemen. Figure S4. Percent change in access to [A] untreated water sources based on SDI, [B] unimproved toilets based on SDI, 2013–2016 in Yemen. (DOCX 1629 kb) [file 12992_2018_354_MOESM2_ESM.docx]

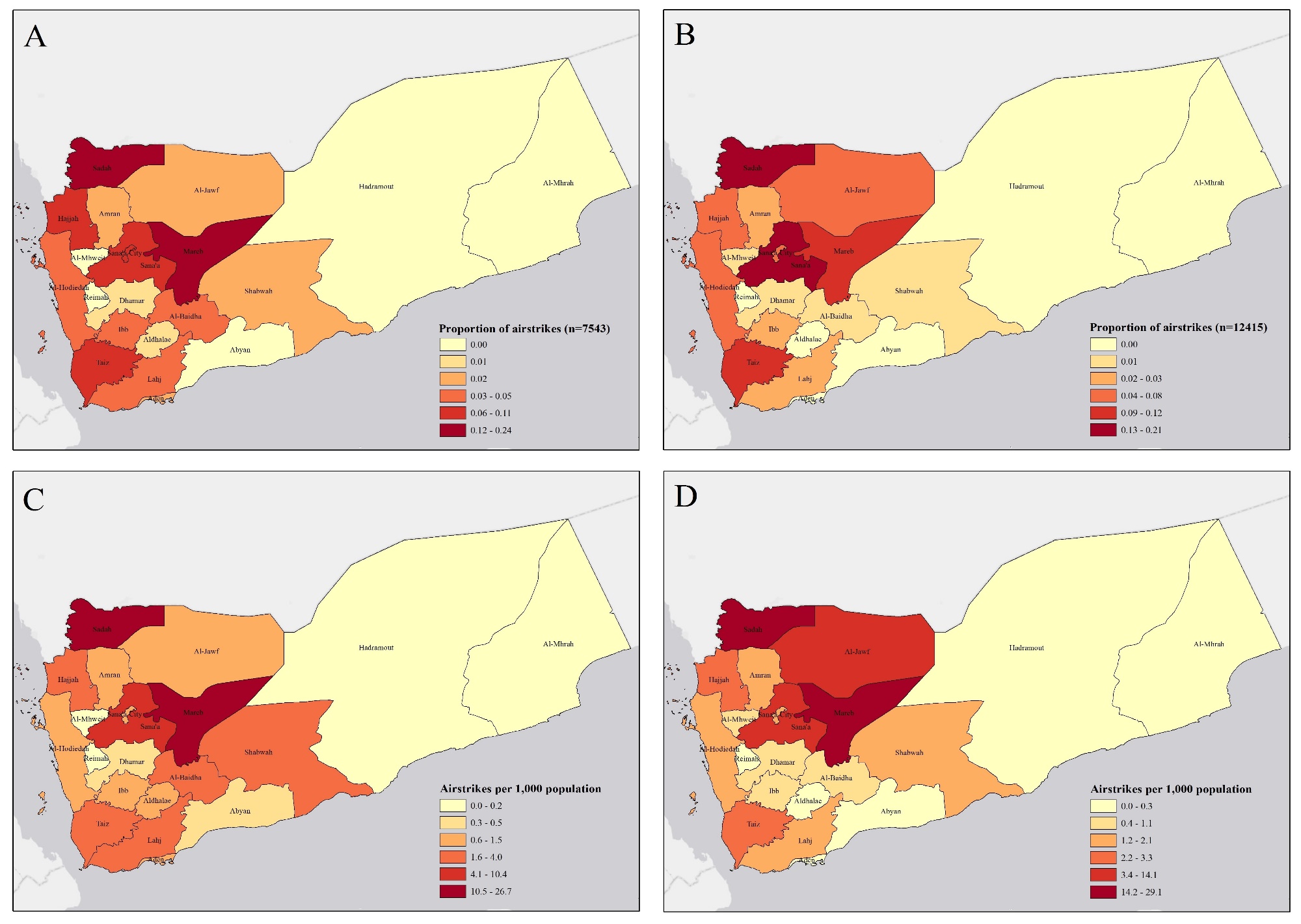


Figure S1: Proportion of total airstrikes in [A] 2015 and [B] 2016, and airstrikes per 1,000 population in [C] 2015 and [D] 2016 in Yemen


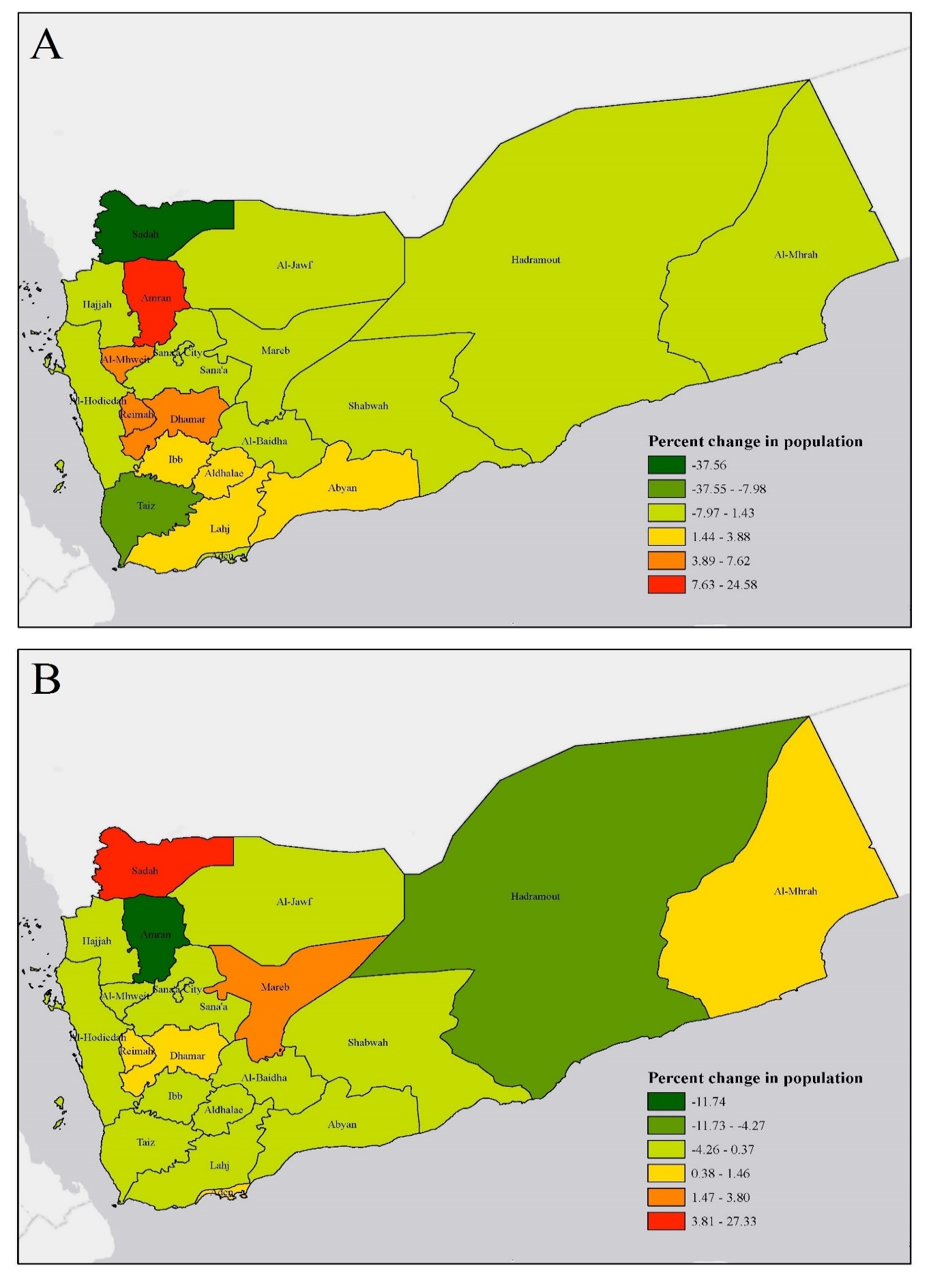


Figure S2: Change in population due to internally displaced persons from [A] 2013 – 2015 and [B] 2015 – 2016 in Yemen


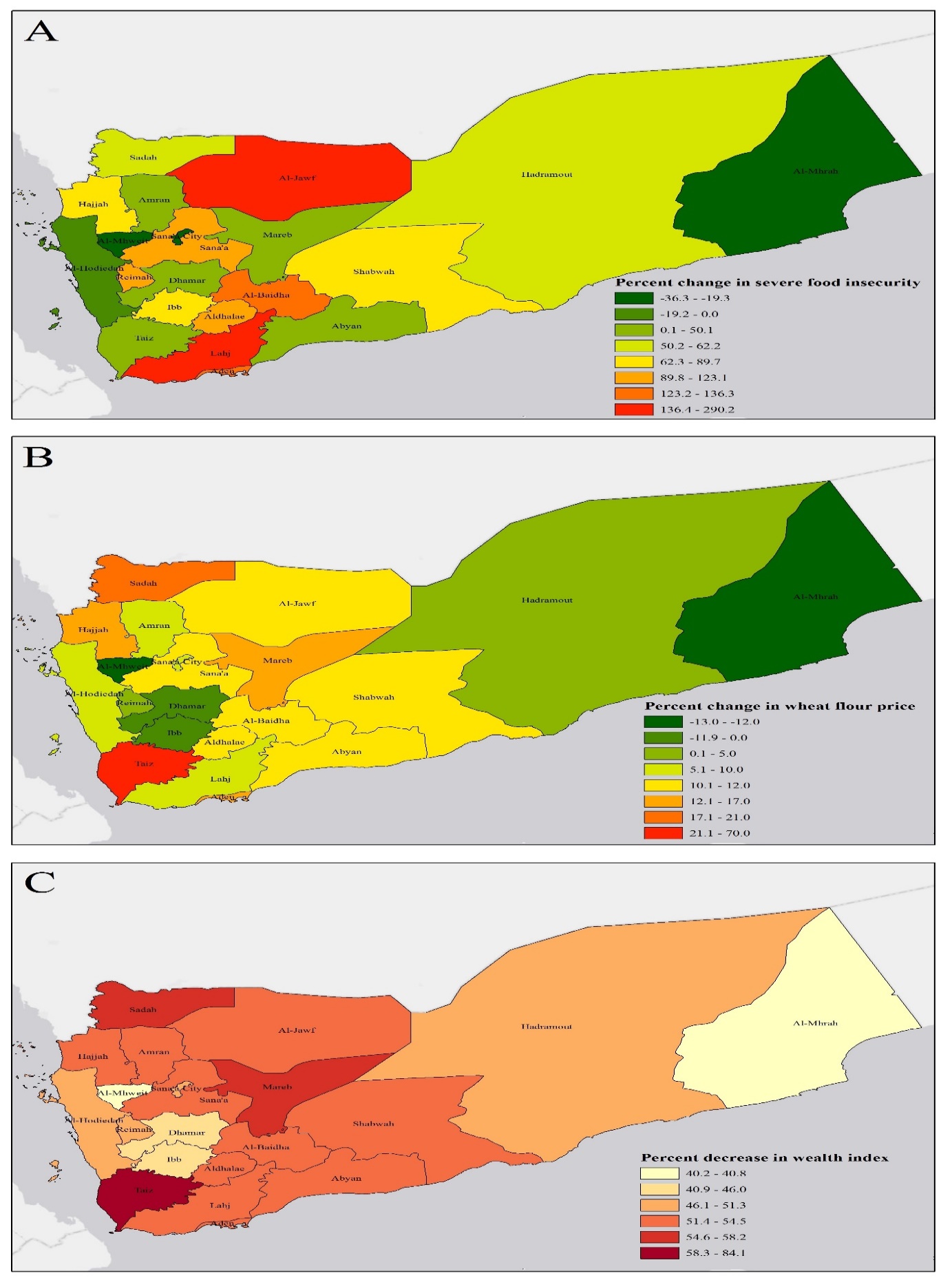


Figure S3: Percent change in [A] severe food insecurity, [B] wheat flour price, [C] wealth index, 2013 – 2016 in Yemen


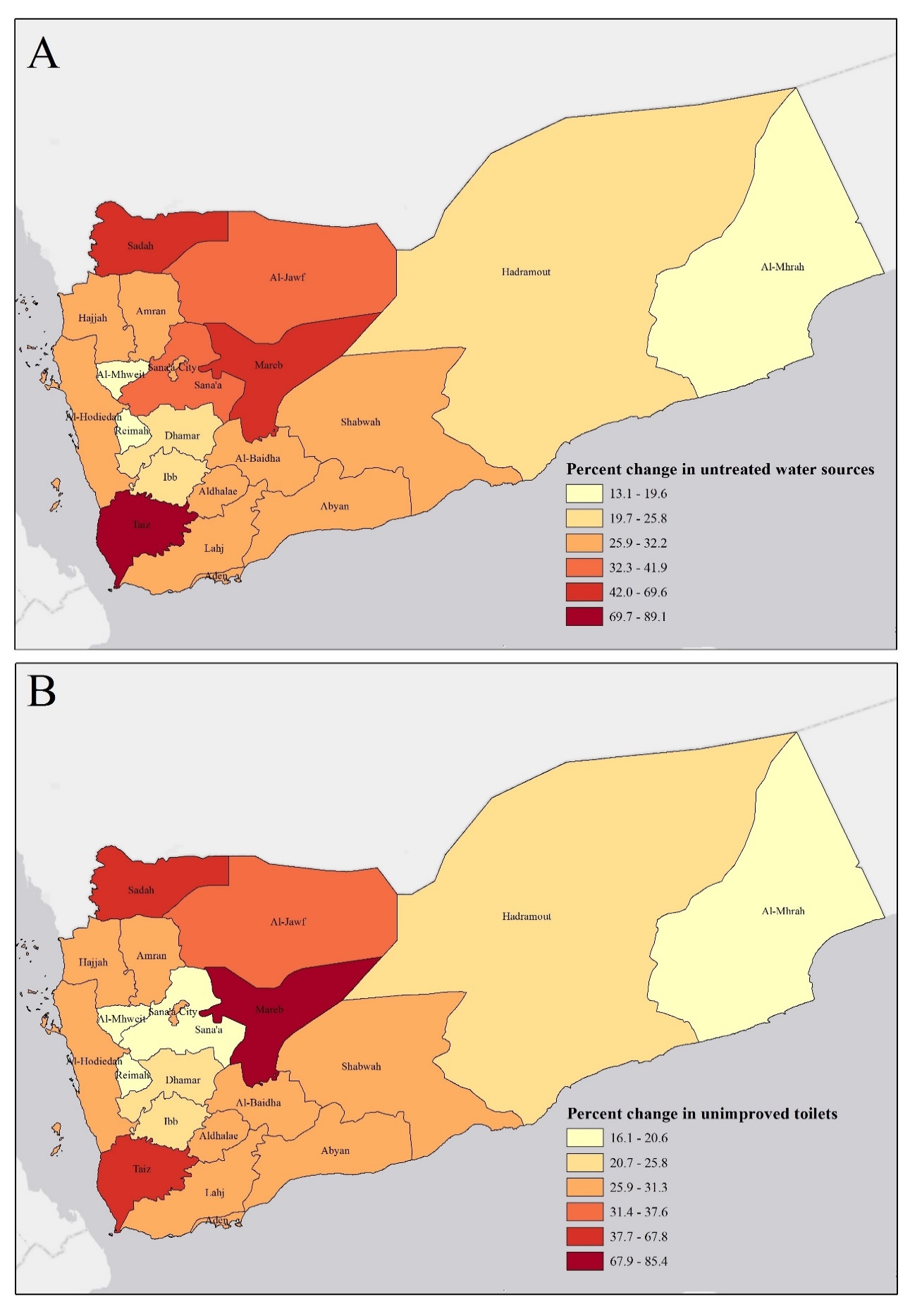


Figure S4: Percent change in access to [A] untreated water sources based on SDI, [B] unimproved toilets based on SDI, 2013 – 2016 in Yemen
