# Supplementary material for: Health in Yemen: losing ground in war time
Source: Global Health. 2018 Apr 25;14:42. doi: 10.1186/s12992-018-0354-9 (PMC5918919; doi:10.1186/s12992-018-0354-9)
Supplement: Supplementary file 3 — Health in Yemen: losing ground in war time, detailed methodology. (DOCX 25 kb) [file 12992_2018_354_MOESM3_ESM.docx]

**Additional file 3**

**Health in Yemen: losing ground in war time, detailed methodology**

**Data review**

We conducted a comprehensive review of all available reports and data in Yemen to assess the impact of the conflict, particularly in key areas of burden. We focused on maternal and child health; infectious diseases such as HIV/AIDS, TB, malaria, polio, pneumonia and diarrhea; nutrition; family planning; and agricultural development.

We also searched for other key priority burden areas, such as mental health and non-communicable diseases, as we expected a similar impact on the disease burden. We reviewed all of the available reports and surveys that focus on disease burden and coverage of related interventions, and which have been conducted from 1990 to present in order to fully understand levels and trends over time, and to align with future GBD efforts as appropriate.

Annex 1 details all data reviewed and their use in this report.

**Data synthesis and estimates production**

We synthesized results to provide updated estimates of selected health outcomes in Yemen for maternal, neonatal, and child health outcomes. Estimates were produced for health conditions for which we had enough quality data.

We summarized estimates (e.g., under-5 mortality, anemia, diarrhea, wasting, mother’s malnutrition, and early childhood immunization) at the national level where they can be produced for all governorates, ensuring these estimates are consistent with those produced by the Global Burden of Disease (GBD). Where possible, we provided estimates at the governorate level. The purpose was to highlight disparities and ensure that national averages do not mask local estimates where the effect of the conflict could be more catastrophic. For indicators not in GBD, we summarized the findings from across the data sources at the governorate level where possible as well. This improved local evidence could be used as an entry point for strategic planning by donors, program partners, and country officials as part of larger long-term surveillance and rebuilding efforts. We presented our estimates both in graphics, including maps, to show how these estimates have changed over time and during the conflict, and in tables.

**Data modeling**

**Indicators estimated**

We estimated trends and annual percentage change for several maternal and child health indicators between 2013 and 2016 by governorate.

For children, we estimated:

- Vaccine coverage, including third-dose Polio (Polio 3), third-dose Penta (Penta 3), third-dose pneumococcal (PCV3), and second-dose measles vaccines among children 12–23 months;
- Global accurate malnutrition (GAM) stunting and wasting, and severe accurate malnutrition (SAM) wasting among children under 5 years;
- Anemia among children aged 6–59 months;
- Under 5 mortality (U5MR).

For women, we modeled:

- Malnutrition;
- Maternal mortality.

**Modeling strategy:**

**Covariates**

- **Conflict indicators:**

To mimic the effect of the conflict on health, we used the changes in economic indicators, access to food, and other variables, including incidence of airstrikes and deaths inflicted by the war. These variables were used as shocks in modeling health indicators.

- - **Airstrikes**

We obtained data on number of airstrikes by governorate for 2015 and 2016 from the Yemen Data Project. To reflect the burden of attacks, we used the number of airstrikes per 1,000 population by governorate.

- - **Access to untreated water and unimproved toilets**

We used the DHS 2013 data to estimate access to untreated water and unimproved toilets. We estimated the change in this access per governorate for 2014, 2015, and 2016 twice. For water, “Well,” “Spring,” “Tanker truck,” “Surface water protected,” and “surface water unprotected” were categorized as untreated. For toilets, “Pit,” “Latrine,” “Bucket,” and “No facility/bush/field” were categorized as unimproved. The untreated observations were summed by governorate and divided by the number of observations in each governorate to get the percent of the population without access to treated water and toilets by governorate.

We assumed access to untreated water and unimproved toilets to have remained the same for 2014 with no special events or incidents having taken place in that year. For 2015–2016, we used yearly change in SDI to estimate change in access to untreated water and unimproved toilets – such that a governorate witnessing a 10% decrease in SDI in that year had an increase in the percent of untreated water and unimproved toilet access by 10% as well.

- - **Internal displacement**

With airstrikes focused on governorates more than the others, many Yemenis were forced to relocate. We used estimates of internally displaced persons (IDP) from the 8^th^ Task Force on Population Movement report for 2015 and the 12^th^ Task Force on Population Movement report for 2016 to adjust the population estimates.

- - **Gross Domestic product (GDP):**

We used changes in GDP growth rate from the Ministry of Planning and Internal Cooperation’s socioeconomic update for 2014, 2015, and 2016. GDP in Yemen decreased by 9.6% between 2013 and 2014, 32.9% between 2014 and 2015, and 12.8% between 2015 and 2016.

- - **Wheat flour prices:**

As wheat flour is an essential ingredient to bread and other foods, we used the change in the 2016 price of wheat flour compared to the pre-crisis period. This change is available from the World Food Program: Yemen market situation update, April 2016.

- - **Severe food insecurity**

With blockades and increased food market prices, food insecurity increased in Yemen. Specifically, we used changes in severe food insecurity from an OCHA and WFP snapshot for 2013, famine early warning system (FEWS) food security outlook for 2014 and 2015, and an Emergency Food Security and Nutrition Assessment (EFSNA) report for 2016 as a predictor for maternal anemia.

- - **Under-5 Casualties**

The number of all-age deaths in 2015 (7,354) was sourced from the International Institute for Strategic Studies (IISS) armed conflict database. The 2016 numbers were sourced from a January 2017 statement from the UN Office for the Coordination of Humanitarian Affairs to the UN Security Council, which listed 7,469 all-age deaths reported from health facilities in 2015 and 2016. The same statement reported only 45% of health facilities still functioning, so the total all-age deaths due to war was 16,598: 7,469/0.45. The number of all-age war deaths from 2015 (7,354) was subtracted from this number to get the number of all-age deaths for 2016 (9,243). The all-age deaths for 2015 and 2016 were then split by age group to get 2,160 and 2,885 under-5 deaths due to war in 2015 and 2016, respectively, which were the numbers used in our under-5 mortality calculations.

- **Under-5 mortality (U5q0)**

First, U5q0 probability from DHS 2013 was converted to a rate using the following formula: rate = ln (1 – probability) / -5. Second, this rate was multiplied by the 2013 population. The 2013 population was calculated by multiplying the proportion of the population in each governorate by the 2013 national under-5 population. The proportion of the population in each governorate was taken from the 2015 pre-crisis population estimates in the 8^th^ report of the Task Force on Population Movement. The 2013 national under-5 population was taken from UN population division estimates. We assumed here that the proportion of the population in each governorate to the total population did not change from 2013 to 2014.

- - **U5q0 2015**

U5q0 rate for 2015 was calculated in five steps. The first two steps are described in the previous section. Third, an additional 2,160 under-5 deaths (taken from the Global Burden of Disease [GBD] estimation framework, discussed above) were distributed by governorates proportionally to the percentage of airstrikes in each governorate in 2015, and added to the total number of child deaths found in the previous step. Data on airstrikes were taken from the Yemen Data Project dataset. Fourth, the total number of child deaths in 2015 was divided by the 2015 population, which was calculated in the same way as in step 2, instead using UN 2015 population estimates and the 8^th^ Report of the Task Force on Population Movement IDP-adjusted population for 2015. Fifth, the 2015 mortality rate from the previous step was converted back to U5q0 probability using the formula from step 1.

- - **U5q0 2016**

Under-5 mortality rate for 2016 was calculated in the same five steps above with several minor differences. The number of added deaths due to war from the GBD estimation framework were 2,885 for 2016, the percentage distribution by governorate of airstrikes from 2016 was used, and population for 2016 was calculated using the IDP-adjusted population for 2016 from the Task Force on Population Movement 12^th^ report.

- **Sociodemographic Indicators:**

We calculated three sociodemographic indices nationally and by governorate, using maternal education, wealth index, and total fertility rate from DHS 2013 data.

- - **Maternal education:**

Maternal education index was calculated for each governorate and nationally, and levels of maternal education taken from the DHS dataset were converted into number of years based on NAFSA, the Association for International Educators, classification. After calculation, the values were scaled between 0 and 1 using min-max scaling.

- - **Fertility index:**

Fertility index was created by taking total fertility rates from the DHS report, scaling, and subtracting from 1 in order for high fertility to correspond with low values.

We assumed that maternal education and fertility rate did not change between years. Hence, maternal education and fertility indices were kept unchanged for the period 2013–2016.

- - **Wealth index:**

Raw wealth index values were also taken from the DHS 2013 report and scaled in the same way as education to create a wealth index. In order to account for the effects of war, we created a wealth index for 2014, 2015, and 2016 using 1) changes in GDP growth rate from the Ministry of Planning and Internal Cooperation’s socioeconomic update for 2014, 2015, and 2016, 2) the number of airstrikes by governorate for 2015 and 2016 from the Yemen Data Project ─there were no airstrikes in 2014 in Yemen─, and 3) the change in wheat flour prices in 2016 ─not available for 2015 and 2014─ relative to the pre-crisis period by governorate from the World Food Program’s Yemen market situation update. The introduction of the additional variables each year is to create a shock effect similar to what the war would cause.

Wealth index for 2014 was estimated by applying a 9.6% decrease in the wealth index from 2013, based on the decrease in GDP from 2013 to 2014. The wealth index for 2015 was calculated in two steps: first, the 2014 wealth index estimates were decreased by 32.9% in all governorates based on the decrease in GDP from 2014 to 2015, and second, these estimates were decreased in each governorate by the number of airstrikes in 2015 divided by the IDP-adjusted population from the 8^th^ Task Force on Population Movement report, multiplied by 100. The wealth index for 2016 was calculated in three steps: first, the 2015 estimates were decreased by 12.8% in all governorates, based on the decrease in GDP from 2015 to 2016. Second, the modified wealth estimates were adjusted in each governorate by the governorate-specific percent changes in wheat flour price relative to the pre-crisis period. Third, these wealth estimates were decreased in each governorate by the number of airstrikes in 2016 divided by the IDP-adjusted population from the 12^th^ Task Force on Population Movement report, multiplied by 100.

- - **Socio-demographic Index**

A Socio-demographic Index (SDI) was calculated for each year and governorate by taking the geometric mean of maternal education, wealth, and fertility indices described above.

**Health indicators**

We used a backward elimination beta-regression ensemble model including SDI split indices and relevant covariates (detailed further) following the general formula:

**Y_2013+t_ = β_0, 2013_ + β_i, 2013_X_i, 2013+t_ + β_j, 2013_X_j, 2013+t_ + ε**

Y: predicted outcome variable

t: number of years from 0 to 3

β: coefficients

X_i_: socio-demographic indices: maternal education, wealth, and fertility

X_j_: covariates such as U5MR

ε: residual

- **Vaccine coverage:**

We received immunization data for all districts and governorates from the Surveillance and Disease Control division of Yemen’s Ministry of Health for the years 2011 through 2016, but Yemen suffers from a weak health information system (health system profile). A formal evaluation of the EPI data does not exist. However, surveillance data for vaccine preventable diseases in the 1980s were shown to have an extremely low sensitivity (prevalence, incidence, and epidemiological features). Administrative data on vaccine coverage were also shown to be much higher than survey data in a 2007 study (immunization coverage and its determinants among children), and were also higher than DHS estimates for 2013. The joint appraisal report for 2016 shows 20%–40% of health data not being recorded at the governorate, district, and health facilities level (Yemen Joint appraisal 2016). Hence, we could not count on EPI data alone, but used these data to adjust DHS estimates and produce the VC for 2014–2016.

Vaccine coverage was estimated through the following six steps:

First, we analyzed the Demographic Health Survey of 2013 for VC at the governorate level.

Second, to produce 2014 VC estimates, and assuming that the trend in administrative data for VC reflects the actual trend in VC, we multiplied the DHS 2013 VC in each governorate by the ratio of VC from administrative data for 2014 over 2013.

Third, we used a similar procedure to produce 2015 VC estimates, with change in administrative VC data between 2015 and 2014. However, in order to account for the movement of internally displaced persons, these new estimates were multiplied by the ratio of 2014 population to 2015 population adjusted for internally displaced persons from the Task Force on Population Movement 8^th^ report. 2014 population was calculated by reducing the Task Force on Population pre-crisis population estimates by 2.6%, the annual population growth given by the UN Population Division. Age distribution of IDP was not available. Hence we used all-age population estimates, assuming an equal age distribution of children between IDPs and the population.

Fourth, a beta-regression model was set up to fit 2015 VC estimates from the previous step with education index, wealth index for 2015, fertility index, administrative VC data from 2015, and under-5 mortality rate for 2015.

Fifth, the coefficients produced in the previous step were used to predict 2016 estimates through a beta-regression model with the same covariates and 2016 values for wealth index, administrative VC data, and under-5 mortality rate. Logged estimates for 2016 were generated by summing the product of each covariate and its associated input. These estimates were then transformed into natural numbers using the formula: e^(estimate)^/(1 + e^(estimate)^) to get the finalized estimates. This process for generating estimates was used in all beta-regression models described hereafter.

Sixth, after a review of the coefficients associated with the inputs used in the beta-regression, wealth index and child mortality were removed from the measles model due to the incorrect directionality of their coefficients; the model implied an increase in wealth would decrease measles coverage and an increase in child mortality would increase measles coverage. New estimates were generated for measles coverage without using wealth and child mortality.

- **Diarrhea:**

We made several unsuccessful attempts at modeling under-5 diarrhea that informed our decision to generate the three sets of estimates described below. We first attempted to calculate diarrhea estimates using cases of reported diarrhea from surveillance sources. These sources were the annual statistical health reports, integrated surveillance yearly reports, and electronic disease early warning system (eDEWS) yearly reports. The annual statistical health reports provide reported diarrhea cases in all governorates in 2013 and five governorates in 2014. Integrated surveillance reports provide reported diarrhea cases in all governorates for 2015 and 2016. The eDEWS yearly reports cover 10 governorates in 2013 and 2014, 16 governorates in 2015, and all governorates in 2016. Integrated surveillance had the highest number of reported cases of under-5 diarrhea (224,479 in 2015, 368,661 in 2016) out of all surveillance sources for years with data in all governorates. This was converted to incidence using the following formulae from the Global Burden of Disease (GBD) study methodology:

Period-prevalence = total under-5 diarrhea cases / under-5 population / 26

Point-prevalence = period-prevalence * duration / (duration + recall period – 1)

Incidence = point-prevalence * 365 / duration

The calculation for under-5 population for 2015 and 2016 was described previously in the U5q0 2015 and 2016 sections. Average duration was 4.3 days and recall period was 15 days.

Integrated surveillance incidence rates for 2015 and 2016 were dramatically lower than the DHS incidence for 2013, with incidence rates of 0.04 and 0.07 per person-year for 2015 and 2016. The DHS incidence rate for 2013 was 6.22 per person-year. We also attempted to model under-5 diarrhea by fitting a beta-regression using the sociodemographic indices and child mortality, which did not have a satisfactory R-squared value. A review of the literature described the lack of access to clean water and toilets as predictors of diarrhea.

- - Estimates based on modeling DHS data

We modeled under-5 diarrhea period-prevalence twice using the two sets of data on untreated water and unimproved toilets detailed in the access to untreated water and unimproved toilets section. A beta-regression was set up to predict the period prevalence of under-5 diarrhea from DHS using maternal education index, wealth index, fertility index, untreated water, unimproved toilet, and under-5 mortality. The coefficients of this model were used to calculate under-5 diarrhea estimates for 2014–2016. The estimates predicted by this model were converted to point-prevalence and incidence of diarrhea using the formulae from the Global Burden of Disease (GBD) methodology described previously.

After reviewing the coefficients, we removed maternal education due to the incorrect directionality of its coefficient; as education increased, diarrhea also increased.

- - Estimates based on GBD 2016 estimates

We calculated under-5 diarrhea in a third approach, using SDI and national estimates of under-5 diarrhea from the GBD 2016 study. In our first attempt, we calculated estimates for each year and governorate using the formula –

Governorate diarrhea point prevalence= national diarrhea (GBD) point prevalence * ((1-governorate SDI) / (1 - national SDI))

This formula produced unexpected decreases in some governorates due to decreases in the national SDI outpacing certain governorate SDIs. We altered our approach to the following two steps:

First, we calculated estimates for each governorate in 2016 using the same formula as above.

Second, we calculated the 2015, 2014, and 2013 estimates backward from 2016 using the following formulae:

Governorate diarrhea point prevalence 2015 = Governorate diarrhea point prevalence 2016 - (Governorate diarrhea point prevalence 2016 * percent change 2015 to 2016)

Percent change = (percent change Governorate SDI / percent change national SDI) * percent change national Diarrhea (GBD)

- **Child nutritional status:**

For indicators of child nutrition, we modeled the percent of moderate and severe under-5 anemia, GAM wasting, SAM wasting, and GAM stunting (as established by WHO standards). For all four indicators, the data from 2013 came from the DHS report. We attempted to model each of these indicators for 2014–2016 using a beta-regression with several combinations of inputs.

For our first attempt, we used maternal education index, wealth index, fertility index, under-5 mortality (U5q0 sections), and diarrhea (see diarrhea; all three diarrhea estimates were tried) as inputs. This produced an unsatisfactory R-squared for all four indicators.

We then tried various combinations of the sociodemographic indices, under-5 mortality, diarrhea, DTP3 coverage (see vaccine coverage), maternal access to health care (from DHS, reported restricted access to a health facility due to money or distance), and food insecurity (Emergency Food Security and Nutrition Assessment, Famine Early Warning System, and UN Office for the Coordination of Human Affairs and World Food Program) with no satisfactory results. We decided to model maternal anemia as a potential input for these models. We then used maternal education index, wealth index, fertility index, under-5 diarrhea, and maternal anemia as inputs in our beta-regression for the four indicators of child nutrition. With three sets of estimates for diarrhea, we generated three models for each indicator. After reviewing the coefficients of the models, we decided on the following as inputs:

Under-5 anemia = maternal education index + wealth index + fertility index + diarrhea + maternal anemia

GAM stunting = maternal education index + wealth index + fertility index + diarrhea

GAM wasting = maternal education index + wealth index + fertility index + maternal anemia

SAM wasting = maternal education index + wealth index + fertility index + maternal anemia

- **Maternal nutritional status:**

For indicators of maternal nutrition we decided to model BMI <18.5, middle upper arm circumference (MUAC) 21–22.9 cm (moderate malnutrition), and MUAC <21cm (acute malnutrition). We set up a beta regression with education index, wealth index, fertility index, and maternal anemia as inputs. The model for BMI was satisfactory, but the MUAC ones were not. We removed maternal anemia from the MUAC models and replaced it with BMI. After review of the coefficients, we also removed wealth index from the MUAC models due to the incorrect directionality of coefficients. The final models are the following:

BMI = education index + wealth index + fertility index + maternal anemia

Moderate MUAC = education index + fertility index + BMI

Severe MUAC = education index + fertility index + BMI

- **Maternal mortality**

We had no additional data pertaining to maternal mortality. Hence, we used GBD 2016 national estimates for maternal mortality and propagated these estimates to the governorate level based on SDI. Then we calculated governorate and national estimates for 2015, 2014, and 2013 based on changes in SDI estimates for these years.
