# Supplementary material for: Health in Yemen: losing ground in war time
Source: Global Health. 2018 Apr 25;14:42. doi: 10.1186/s12992-018-0354-9 (PMC5918919; doi:10.1186/s12992-018-0354-9)
Supplement: Supplementary file 4 — Estimates of maternal and child health indicators and their 95% confidence intervals, 2013–2016, and percent change from 2013 to 2016 by governorate, Yemen. (DOCX 80 kb) [file 12992_2018_354_MOESM4_ESM.docx]

Table S1: Diphtheria-Tetanus-Pertussis 3^rd^ dose vaccine coverage and 95% confidence intervals, 2013 - 2016, and percent change from 2013 to 2016 among children aged 12-23 months by governorate, Yemen

| **Governorate** | **2013 (95% CI)** | **2014*** | **2015 (95% CI)** | **2016 (95% CI)** | **% change 2013 to 2016** |
| --- | --- | --- | --- | --- | --- |
| Abyan | 50.6 (36.7 - 64.4) | 49.2 | 61.6 (52.5 - 69.7) | 59.4 (46.1 - 70.3) | 17.5 |
| Aden | 72.9 (63.4 - 82.4) | 72.5 | 61.6 (46.5 - 74.5) | 57.8 (40.8 - 73.0) | -20.8 |
| Al-Baidha | 34.5 (24.0 - 45.0) | 34.5 | 47.5 (35.3 - 56.0) | 45.6 (32.2 - 54.0) | 32.1 |
| Aldhalae | 42.0 (27.6 - 56.5) | 41.2 | 50.9 (43.1 - 58.4) | 48.9 (40.2 - 57.8) | 16.4 |
| Al-Hodeida | 56.3 (46.0 - 66.6) | 56.9 | 52.6 (36.1 - 62.4) | 51.8 (34.0 - 63.5) | -8.0 |
| Al-Jawf | 20.8 (9.2 - 32.4) | 20.9 | 24.7 (14.1 - 71.0) | 23.0 (13.5 - 67.5) | 10.6 |
| Al-Mahrah | 75.1 (66.0 - 84.1) | 75.8 | 56.0 (38.0 - 69.1) | 55.9 (38.5 - 68.6) | -25.6 |
| Al-Mahwit | 62.4 (51.7 - 73.1) | 66.7 | 56.3 (46.4 - 65.0) | 56.1 (46.5 - 65.0) | -10.1 |
| Amran | 62.6 (51.7 - 73.5) | 65.8 | 50.3 (38.1 - 62.7) | 48.5 (37.1 - 59.6) | -22.5 |
| Dhamar | 57.3 (48.0 - 66.6) | 56.6 | 56.6 (40.4 - 69.5) | 56.1 (41.7 - 68.1) | -2.0 |
| Hadramout | 77.5 (65.2 - 89.7) | 77.9 | 60.6 (48.4 - 69.1) | 58.3 (45.3 - 67.7) | -24.8 |
| Hajjah | 49.1 (36.4 - 61.8) | 49.0 | 43.8 (25.0 - 58.8) | 43.7 (24.9 - 58.8) | -10.9 |
| Ibb | 50.9 (39.7 - 62.2) | 49.9 | 64.8 (53.7 - 71.9) | 64.1 (52.5 - 71.3) | 25.9 |
| Lahj | 73.1 (63.2 - 82.9) | 73.3 | 51.9 (41.3 - 64.9) | 50.2 (35.7 - 66.2) | -31.4 |
| Mareb | 41.9 (30.0 - 53.7) | 42.9 | 45.1 (30.9 - 63.1) | 42.9 (24.5 - 61.4) | 2.4 |
| Reimah | 61.0 (49.6 - 72.4) | 62.7 | 58.6 (41.2 - 68.5) | 58.6 (40.9 - 68.7) | -3.9 |
| Sadah | 20.0 (12.1 - 27.8) | 19.9 | 17.1 ( 5.0 - 28.9) | 16.4 ( 5.4 - 32.3) | -17.9 |
| Sana’a | 51.4 (39.7 - 63.1) | 49.1 | 40.0 (30.0 - 47.2) | 37.1 (25.9 - 45.7) | -27.7 |
| Sana’a City | 88.1 (81.9 - 94.4) | 91.4 | 78.9 (60.8 - 87.9) | 76.3 (59.7 - 86.6) | -13.4 |
| Shabwah | 48.5 (35.7 - 61.2) | 47.0 | 49.1 (32.1 - 63.4) | 46.0 (32.9 - 56.3) | -5.2 |
| Taiz | 75.7 (67.5 - 84.0) | 76.7 | 48.8 (35.1 - 61.3) | 43.4 (19.4 - 67.8) | -42.6 |
| National | 59.6 (56.8 - 62.5) | 59.6 | 52.0 (44.9 - 56.9) | 49.9 (38.9 - 56.8) | -16.2 |

*2014 estimates were produced based only on an administrative vaccine data correction factor and 95% CI were not computed

Table S2: Measles 1^st^ dose vaccine coverage and 95% confidence intervals, 2013 - 2016, and percent change from 2013 to 2016 among children aged 12-23 months by governorate, Yemen

| **Governorate** | **2013 (95% CI)** | **2014*** | **2015 (95% CI)** | **2016 (95% CI)** | **% change 2013 to 2016** |
| --- | --- | --- | --- | --- | --- |
| Abyan | 49.6 (37.5 - 61.8) | 42.6 | 50.9 (44.9 - 55.2) | 55.1 (48.6 – 60.4) | 11.1 |
| Aden | 79.8 (73.0 - 86.7) | 76.6 | 45.3 (35.9 - 52.0) | 50.7 (41.0 – 57.2) | -36.4 |
| Al-Baidha | 41.5 (31.4 - 51.6) | 33.9 | 42.8 (33.5 - 49.0) | 49.6 (41.8 – 55.4) | 19.5 |
| Aldhalae | 52.2 (39.8 - 64.7) | 43.1 | 44.7 (39.9 - 48.1) | 56.1 (51.5 – 59.7) | 7.5 |
| Al-Hodeida | 65.2 (55.2 - 75.1) | 54.0 | 56.3 (49.4 - 61.2) | 57.1 (50.1 – 62.1) | -12.4 |
| Al-Jawf | 29.3 (16.6 - 41.9) | 24.9 | 25.0 (16.7 - 38.8) | 26.4 (18.0 – 40.4) | -9.9 |
| Al-Mahrah | 71.1 (58.2 - 84.0) | 61.3 | 52.2 (46.7 - 56.9) | 56.6 (50.2 – 62.5) | -20.4 |
| Al-Mahwit | 69.4 (59.3 - 79.5) | 66.3 | 56.6 (49.3 - 63.8) | 50.5 (44.7 – 56.2) | -27.3 |
| Amran | 63.2 (52.8 - 73.7) | 55.1 | 45.4 (40.1 - 50.7) | 45.3 (40.1 – 50.6) | -28.4 |
| Dhamar | 65.7 (56.5 - 74.9) | 54.2 | 53.3 (46.2 - 58.8) | 51.4 (44.8 – 56.8) | -21.7 |
| Hadramout | 62.8 (51.9 - 73.7) | 49.0 | 53.8 (47.3 - 58.0) | 51.3 (44.9 – 55.6) | -18.3 |
| Hajjah | 56.2 (43.5 - 68.9) | 43.7 | 46.9 (38.9 - 53.3) | 50.9 (43.4 – 57.4) | -9.4 |
| Ibb | 59.1 (49.2 - 68.9) | 49.5 | 61.1 (54.9 - 67.3) | 58.4 (53.0 – 63.5) | -1.2 |
| Lahj | 68.9 (56.2 - 81.6) | 58.3 | 44.7 (40.0 - 48.3) | 53.8 (49.0 – 58.1) | -21.9 |
| Mareb | 53.6 (41.2 - 66.0) | 47.4 | 45.6 (41.5 - 49.2) | 56.7 (51.4 – 62.2) | 5.8 |
| Reimah | 67.7 (57.7 - 77.8) | 58.9 | 60.5 (52.4 - 67.3) | 58.6 (50.9 – 64.6) | -13.5 |
| Sadah | 44.3 (32.7 - 55.9) | 47.4 | 27.5 (13.1 - 38.2) | 41.7 (26.4 – 52.0) | -6.0 |
| Sana’a | 59.1 (49.5 - 68.6) | 52.6 | 46.9 (41.6 - 51.0) | 45.0 (39.3 – 49.1) | -23.9 |
| Sana’a City | 76.1 (69.9 - 82.3) | 69.7 | 63.7 (52.0 - 73.6) | 62.8 (51.3 – 72.4) | -17.4 |
| Shabwah | 48.8 (36.2 - 61.5) | 37.3 | 43.0 (33.4 - 49.5) | 48.1 (39.5 – 54.2) | -1.5 |
| Taiz | 74.0 (67.4 - 80.6) | 62.5 | 44.5 (38.6 - 48.7) | 49.0 (43.8 – 52.7) | -33.7 |
| National | 63.3 (60.7 - 65.9) | 53.9 | 44.5 (39.0 - 48.4) | 52.0 (47.8 – 55.4) | -17.9 |

*2014 estimates were produced based only on an administrative vaccine data correction factor and 95% CI were not computed

Table S3: Pneumococcal vaccine coverage and 95% confidence intervals, 2013 - 2016, and percent change from 2013 to 2016 among children aged 12-23 months by governorate, Yemen

| **Governorate** | **2013 (95% CI)** | **2014*** | **2015 (95% CI)** | **2016 (95% CI)** | **% change 2013 to 2016** |
| --- | --- | --- | --- | --- | --- |
| Abyan | 31.7 (20.6 - 42.8) | 30.8 | 40.0 (33.3 - 44.7) | 39.9 (32.1 - 46.5) | 25.8 |
| Aden | 58.9 (48.4 - 69.4) | 58.5 | 38.0 (27.1 - 43.7) | 38.1 (27.4 - 46.6) | -35.4 |
| Al-Baidha | 19.7 (13.4 - 26.0) | 19.7 | 28.7 (21.6 - 33.7) | 28.9 (21.6 - 33.8) | 46.8 |
| Aldhalae | 23.6 (10.5 - 36.7) | 20.5 | 33.7 (28.1 - 38.3) | 33.8 (27.8 - 39.0) | 43.1 |
| Al-Hodeida | 38.1 (28.5 - 47.8) | 38.5 | 33.9 (24.7 - 39.2) | 33.9 (24.3 - 40.3) | -10.9 |
| Al-Jawf | 8.9 (2.2 - 15.5) | 5.9 | 29.3 (19.7 - 57.5) | 27.8 (18.9 - 54.2) | 212.7 |
| Al-Mahrah | 41.2 (28.6 - 53.7) | 41.6 | 34.4 (24.1 - 41.7) | 34.5 (24.5 - 41.6) | -16.2 |
| Al-Mahwit | 47.7 (37.8 - 57.7) | 51.0 | 37.2 (30.9 - 43.6) | 37.1 (30.8 - 43.5) | -22.2 |
| Amran | 39.0 (29.3 - 48.6) | 41.0 | 33.3 (27.1 - 41.8) | 32.1 (26.5 - 39.5) | -17.7 |
| Dhamar | 34.6 (25.8 - 43.4) | 34.2 | 35.3 (28.1 - 45.0) | 35.4 (28.6 - 44.2) | 2.4 |
| Hadramout | 42.7 (30.9 - 54.5) | 42.9 | 38.9 (30.0 - 43.6) | 38.7 (29.8 - 44.3) | -9.5 |
| Hajjah | 35.6 (23.5 - 47.6) | 35.5 | 33.0 (21.6 - 40.8) | 32.9 (21.5 - 40.8) | -7.5 |
| Ibb | 34.6 (23.0 - 46.3) | 33.9 | 40.8 (34.3 - 45.8) | 40.9 (34.2 - 46.0) | 18.3 |
| Lahj | 55.0 (43.4 - 66.6) | 55.2 | 36.8 (29.6 - 45.1) | 36.9 (28.1 - 46.8) | -33.0 |
| Mareb | 27.6 (18.6 - 36.7) | 28.2 | 28.3 (21.7 - 40.2) | 29.0 (21.5 - 41.4) | 5.2 |
| Reimah | 39.8 (28.4 - 51.3) | 40.9 | 39.8 (30.1 - 47.0) | 40.0 (30.1 - 47.3) | 0.6 |
| Sadah | 12.7 (5.4 - 20.0) | 12.7 | 12.6 (5.4 - 20.3) | 14.0 (6.7 - 22.2) | 10.6 |
| Sana’a | 38.5 (27.1 - 49.8) | 36.8 | 26.3 (20.4 - 30.8) | 25.1 (19.2 - 30.5) | -34.9 |
| Sana’a City | 54.2 (45.9 - 62.4) | 56.3 | 50.5 (36.8 - 59.0) | 50.3 (37.5 - 60.1) | -7.2 |
| Shabwah | 36.9 (25.8 - 48.0) | 35.7 | 30.8 (19.8 - 36.9) | 30.7 (21.7 - 36.2) | -16.7 |
| Taiz | 42.8 (33.9 - 51.8) | 43.4 | 32.3 (25.0 - 39.2) | 31.9 (20.4 - 45.8) | -25.4 |
| National | 38.4 (35.6 - 41.3) | 38.3 | 33.5 (28.7 - 36.3) | 33.4 (27.0 - 37.0) | -13.1 |

*2014 estimates were produced based only on an administrative vaccine data correction factor and 95% CI were not computed

Table S4: Polio 3^rd^ dose vaccine coverage and 95% confidence intervals, 2013 - 2016, and percent change from 2013 to 2016 among children aged 12-23 months by governorate, Yemen

| **Governorate** | **2013 (95% CI)** | **2014*** | **2015 (95% CI)** | **2016 (95% CI)** | **% change 2013 to 2016** |
| --- | --- | --- | --- | --- | --- |
| Abyan | 55.9 (44.2 - 67.7) | 54.3 | 58.7 (53.8 - 64.2) | 58.2 (50.6 - 66.1) | 4.1 |
| Aden | 75.4 (65.9 - 84.8) | 75.8 | 56.4 (47.7 - 63.8) | 55.4 (45.6 - 66.3) | -26.5 |
| Al-Baidha | 38.1 (28.7 - 47.5) | 38.1 | 47.8 (38.6 - 53.6) | 47.3 (37.4 - 54.0) | 24.1 |
| Aldhalae | 44.1 (30.0 - 58.1) | 44.1 | 48.1 (42.8 - 53.8) | 47.6 (42.6 - 54.7) | 7.8 |
| Al-Hodeida | 58.9 (47.5 - 70.3) | 59.6 | 54.5 (44.7 - 61.3) | 54.3 (43.5 - 62.1) | -7.9 |
| Al-Jawf | 20.2 (10.0 - 30.4) | 20.1 | 23.3 (18.4 - 60.9) | 22.9 (17.2 - 59.0) | 13.4 |
| Al-Mahrah | 59.2 (44.1 - 74.2) | 59.8 | 49.9 (38.8 - 57.2) | 49.9 (39.3 - 57.2) | -15.8 |
| Al-Mahwit | 62.5 (53.9 - 71.2) | 66.8 | 54.7 (49.4 - 60.5) | 54.7 (49.2 - 60.6) | -12.5 |
| Amran | 63.5 (55.4 - 71.6) | 66.8 | 47.4 (39.5 - 54.7) | 47.0 (40.0 - 52.8) | -26.0 |
| Dhamar | 55.4 (46.3 - 64.5) | 54.7 | 53.9 (44.3 - 60.7) | 53.7 (45.3 - 60.1) | -3.0 |
| Hadramout | 68.3 (56.9 - 79.8) | 68.6 | 56.0 (47.9 - 61.8) | 55.4 (47.6 - 62.0) | -18.9 |
| Hajjah | 47.6 (36.4 - 58.8) | 47.5 | 45.9 (36.3 - 56.5) | 45.9 (36.4 - 56.4) | -3.7 |
| Ibb | 60.0 (50.9 - 69.0) | 58.9 | 62.6 (56.5 - 67.1) | 62.4 (56.4 - 67.0) | 4.0 |
| Lahj | 71.7 (62.3 - 81.1) | 71.9 | 49.4 (43.4 - 60.6) | 48.9 (41.2 - 62.1) | -31.7 |
| Mareb | 45.8 (34.8 - 56.8) | 46.8 | 47.7 (38.4 - 61.8) | 47.1 (35.2 - 61.6) | 2.9 |
| Reimah | 52.8 (41.5 - 64.0) | 54.5 | 59.0 (52.2 - 66.0) | 59.0 (52.1 - 66.2) | 11.7 |
| Sadah | 21.5 (12.7 - 30.4) | 21.4 | 21.1 (9.5 - 32.1) | 20.9 (10.0 - 31.6) | -2.9 |
| Sana’a | 51.4 (39.7 - 63.1) | 49.2 | 40.2 (33.0 - 45.4) | 39.5 (31.3 - 46.8) | -23.2 |
| Sana’a City | 73.7 (65.7 - 81.7) | 76.5 | 73.8 (65.8 - 80.9) | 73.0 (63.9 - 80.6) | -0.9 |
| Shabwah | 45.4 (33.4 - 57.5) | 43.9 | 44.9 (33.1 - 54.8) | 44.1 (35.1 - 52.1) | -2.9 |
| Taiz | 71.4 (63.1 - 79.7) | 72.3 | 49.6 (41.2 - 59.0) | 48.2 (33.4 - 65.5) | -32.5 |
| National | 58.7 (55.9 - 61.5) | 58.7 | 50.8 (46.5 - 54.3) | 50.3 (44.3 - 55.4) | -14.3 |

*2014 estimates were produced based only on an administrative vaccine data correction factor and 95% CI were not computed

Table S5: Diarrheal disease incidence and 95% confidence intervals, 2013 - 2016, and percent change from 2013 to 2016 among children under five by governorate, Yemen

| **Governorate** | **2013 (95% CI)** | **2014 (95% CI)** | **2015 (95% CI)** | **2016 (95% CI)** | **% change 2013 to 2016** |
| --- | --- | --- | --- | --- | --- |
| Abyan | 5.2 (4.4 - 6.0) | 5.9 (5.1 - 6.7) | 6.1 (4.6 - 7.9) | 6.3 (4.5 - 8.6) | 21.9 |
| Aden | 3.8 (3.1 - 4.6) | 4.0 (3.0 - 5.5) | 4.1 (2.3 - 6.4) | 4.1 (1.8 - 7.0) | 5.9 |
| Al-Baidha | 8.0 (6.5 - 9.5) | 7.3 (5.0 - 9.2) | 7.8 (5.0 - 10.2) | 8.0 (4.8 - 10.8) | 0.5 |
| Aldhalae | 7.1 (6.3 - 7.9) | 6.1 (5.2 - 6.9) | 6.5 (5.0 - 8.1) | 6.7 (5.0 - 8.8) | 16.9 |
| Al-Hodeida | 5.7 (4.7 - 6.8) | 6.0 (4.5 - 8.2) | 6.2 (4.6 - 8.5) | 6.3 (4.7 - 8.7) | -11.1 |
| Al-Jawf | 7.1 (5.6 - 8.6) | 5.3 (2.5 - 7.1) | 5.7 (2.8 - 7.9) | 6.6 (4.0 - 9.0) | 71.8 |
| Al-Mahrah | 3.8 (2.7 - 5.0) | 4.9 (3.7 - 6.3) | 5.0 (2.8 - 6.8) | 4.9 (2.7 - 6.8) | -42.6 |
| Al-Mahwit | 8.6 (7.5 - 9.7) | 7.8 (6.6 - 9.1) | 8.1 (6.7 - 9.6) | 8.1 (6.7 - 9.6) | 14.4 |
| Amran | 7.2 (6.4 - 7.9) | 7.5 (6.4 - 9.3) | 7.3 (6.0 - 8.9) | 8.1 (6.8 - 9.8) | 13.1 |
| Dhamar | 6.2 (5.1 - 7.2) | 6.8 (5.8 - 9.3) | 7.0 (5.9 - 8.9) | 7.1 (6.0 - 8.8) | 14.3 |
| Hadramout | 2.7 (2.1 - 3.4) | 4.5 (3.2 - 5.7) | 4.6 (2.5 - 6.7) | 4.7 (2.3 - 7.4) | 72.8 |
| Hajjah | 5.9 (5.2 - 6.5) | 6.7 (4.3 - 9.3) | 7.3 (5.3 - 9.8) | 7.8 (5.7 - 10.3) | 32.8 |
| Ibb | 7.5 (6.6 - 8.5) | 6.1 (5.0 - 7.2) | 6.3 (4.7 - 7.9) | 6.4 (4.6 - 8.1) | -15.5 |
| Lahj | 5.2 (4.1 - 6.3) | 5.6 (4.5 - 7.2) | 6.0 (4.7 - 7.7) | 6.3 (4.7 - 8.3) | 20.4 |
| Mareb | 6.7 (5.4 - 8.0) | 6.9 (5.6 - 8.2) | 9.7 (5.7 - 13.0) | 10.0 (6.2 - 13.4) | 48.6 |
| Reimah | 6.1 (4.9 - 7.3) | 8.2 (6.8 - 10.5) | 8.4 (6.8 - 10.6) | 8.3 (6.7 - 10.6) | 36.7 |
| Sadah | 7.0 (6.1 - 7.9) | 6.5 (5.5 - 7.5) | 10.0 (6.0 - 14.7) | 9.9 (6.3 - 13.7) | 41.2 |
| Sana’a | 6.8 (5.8 - 7.8) | 7.3 (6.1 - 9.8) | 7.8 (6.3 - 10.5) | 8.6 (6.8 - 11.4) | 27.1 |
| Sana’a City | 4.8 (3.8 - 5.7) | 4.2 (3.3 - 5.6) | 4.4 (2.5 - 6.5) | 4.5 (2.1 - 7.2) | -6.0 |
| Shabwah | 5.3 (4.1 - 6.5) | 5.5 (4.6 - 6.3) | 5.9 (4.6 - 7.1) | 6.1 (4.4 - 8.0) | 15.1 |
| Taiz | 6.9 (6.2 - 7.6) | 6.4 (5.4 - 7.3) | 7.0 (5.6 - 8.6) | 8.4 (6.0 - 10.8) | 21.4 |
| National | 6.2 (6.0 - 6.5) | 6.1 (5.4 - 6.9) | 6.6 (5.4 - 8.1) | 7.0 (5.5 - 8.9) | 11.9 |

Table S6: Global acute malnutrition-stunting prevalence and 95% confidence intervals, 2013 - 2016, and percent change from 2013 to 2016 among children under five by governorate, Yemen

| **Governorate** | **2013 (95% CI)** | **2014 (95% CI)** | **2015 (95% CI)** | **2016 (95% CI)** | **% change 2013 to 2016** |
| --- | --- | --- | --- | --- | --- |
| Abyan | 23.4 (18.9 - 27.9) | 38.2 (32.3 - 43.4) | 43.3 (34.3 - 50.7) | 45.8 (34.8 - 54.7) | 95.9 |
| Aden | 23.7 (18.7 - 28.8) | 23.4 (15.9 - 29.4) | 29.8 (19.8 - 40.0) | 33.1 (20.6 - 45.9) | 39.6 |
| Al-Baidha | 35.6 (28.9 - 42.3) | 44.7 (37.4 - 51.7) | 50.1 (41.0 - 58.3) | 52.5 (42.1 - 61.7) | 47.5 |
| Aldhalae | 51.9 (46.6 - 57.1) | 43.0 (39.8 - 45.8) | 48.0 (42.6 - 52.4) | 50.5 (43.4 - 55.9) | 4.6 |
| Al-Hodeida | 48.6 (43.8 - 53.5) | 50.9 (43.9 - 57.0) | 53.2 (45.1 - 60.3) | 54.3 (45.2 - 62.1) | 15.8 |
| Al-Jawf | 57.1 (47.4 - 66.8) | 50.2 (39.6 - 59.1) | 53.1 (41.9 - 61.8) | 56.3 (44.3 - 64.6) | -32.3 |
| Al-Mahrah | 23.1 (16.3 - 29.9) | 32.3 (26.6 - 38.1) | 38.5 (32.2 - 43.4) | 38.6 (32.3 - 43.6) | 160.0 |
| Al-Mahwit | 54.9 (49.5 - 60.2) | 57.6 (52.7 - 62.0) | 59.8 (53.8 - 64.9) | 60.1 (54.1 - 65.2) | -8.0 |
| Amran | 57.7 (53.9 - 61.6) | 57.6 (50.5 - 62.7) | 59.5 (53.8 - 64.0) | 62.0 (56.7 - 67.4) | 7.5 |
| Dhamar | 59.2 (54.7 - 63.7) | 56.6 (49.1 - 61.3) | 59.1 (52.8 - 63.3) | 59.8 (53.7 - 64.0) | 1.0 |
| Hadramout | 30.1 (25.5 - 34.6) | 30.4 (23.9 - 35.0) | 36.2 (27.9 - 43.6) | 38.5 (29.5 - 47.6) | 27.9 |
| Hajjah | 58.8 (53.1 - 64.4) | 62.3 (55.3 - 68.7) | 63.5 (56.6 - 70.0) | 64.3 (57.1 - 71.3) | 9.4 |
| Ibb | 47.3 (41.9 - 52.7) | 46.0 (42.8 - 48.7) | 50.0 (44.4 - 54.3) | 50.9 (44.5 - 55.8) | 7.6 |
| Lahj | 37.9 (31.4 - 44.3) | 43.2 (38.0 - 47.4) | 47.8 (39.7 - 54.1) | 49.9 (40.2 - 57.3) | 31.7 |
| Mareb | 41.3 (34.5 - 48.1) | 46.8 (42.2 - 50.7) | 58.2 (45.3 - 68.3) | 61.3 (46.1 - 72.1) | 48.5 |
| Reimah | 62.7 (57.0 - 68.4) | 64.3 (58.3 - 69.7) | 64.9 (58.8 - 70.8) | 64.8 (58.7 - 70.6) | 3.4 |
| Sadah | 58.5 (51.1 - 65.8) | 49.0 (38.1 - 56.9) | 60.6 (44.6 - 74.5) | 62.5 (45.6 - 75.7) | 6.9 |
| Sana’a | 47.8 (42.5 - 53.1) | 51.3 (46.7 - 55.8) | 55.8 (50.0 - 61.2) | 59.5 (51.5 - 66.7) | 24.4 |
| Sana’a City | 30.7 (26.4 - 34.9) | 23.5 (16.0 - 29.4) | 30.3 (20.2 - 39.8) | 33.4 (21.3 - 45.5) | 8.9 |
| Shabwah | 28.6 (22.7 - 34.5) | 37.0 (31.1 - 44.9) | 43.6 (37.4 - 48.6) | 47.0 (39.4 - 53.2) | 64.4 |
| Taiz | 46.8 (42.8 - 50.7) | 43.3 (35.2 - 50.1) | 48.0 (36.1 - 56.9) | 55.7 (38.4 - 67.7) | 18.9 |
| National | 46.5 (45.1 - 48.0) | 44.9 (40.9 - 48.0) | 49.7 (43.1 - 54.8) | 52.3 (44.0 - 58.5) | 12.4 |

Table S7: Moderate and severe anemia prevalence and 95% confidence intervals, 2013 - 2016, and percent change from 2013 to 2016 among children under five by governorate, Yemen

| **Governorate** | **2013*** | **2014 (95% CI)** | **2015 (95% CI)** | **2016 (95% CI)** | **% change 2013 to 2016** |
| --- | --- | --- | --- | --- | --- |
| Abyan | 92.7 | 80.5 (73.1 - 86.2) | 67.9 (54.8 - 79.3) | 97.4 (91.6 - 99.2) | 5.0 |
| Aden | 81.1 | 95.9 (91.3 - 98.2) | 97.6 (92.9 - 99.2) | 97.6 (91.9 - 99.4) | 20.3 |
| Al-Baidha | 69.0 | 74.1 (59.7 - 79.8) | 66.5 (50.0 - 76.9) | 87.1 (73.7 - 93.8) | 26.2 |
| Aldhalae | 68.4 | 66.5 (60.2 - 70.2) | 70.0 (63.0 - 76.7) | 86.1 (78.3 - 91.6) | 25.9 |
| Al-Hodeida | 81.6 | 79.3 (70.2 - 86.0) | 91.5 (85.1 - 95.6) | 96.3 (91.5 - 98.5) | 18.0 |
| Al-Jawf | 93.1 | 63.9 (46.3 - 80.6) | 94.1 (83.4 - 97.3) | 79.6 (60.8 - 89.6) | -14.5 |
| Al-Mahrah | 80.2 | 45.9 (29.9 - 58.0) | 78.8 (71.0 - 83.6) | 97.2 (93.2 - 98.8) | 21.2 |
| Al-Mahwit | 85.7 | 70.5 (59.6 - 77.6) | 80.2 (68.6 - 86.9) | 73.5 (61.7 - 81.3) | -14.2 |
| Amran | 82.3 | 63.9 (56.1 - 74.1) | 90.2 (82.8 - 94.4) | 73.8 (65.3 - 80.5) | -10.3 |
| Dhamar | 68.4 | 73.7 (67.2 - 82.7) | 82.3 (75.9 - 88.2) | 76.2 (69.8 - 83.1) | 11.5 |
| Hadramout | 78.1 | 55.0 (37.4 - 64.1) | 73.7 (59.1 - 83.0) | 74.1 (57.4 - 84.9) | -5.2 |
| Hajjah | 82.3 | 85.3 (78.1 - 90.1) | 87.0 (79.9 - 91.8) | 91.9 (84.9 - 95.7) | 11.6 |
| Ibb | 55.6 | 61.9 (52.4 - 67.9) | 67.0 (57.2 - 74.8) | 67.6 (57.5 - 76.2) | 21.5 |
| Lahj | 92.3 | 97.2 (93.0 - 98.8) | 88.7 (80.0 - 93.8) | 97.2 (91.9 - 99.0) | 5.4 |
| Mareb | 67.6 | 85.9 (77.8 - 90.7) | 95.5 (84.9 - 98.8) | 79.7 (59.4 - 91.7) | 17.8 |
| Reimah | 72.9 | 75.0 (65.8 - 82.4) | 90.3 (81.4 - 95.0) | 96.6 (90.7 - 98.8) | 32.5 |
| Sadah | 92.3 | 98.6 (94.7 - 99.6) | 88.8 (64.7 - 95.1) | 94.9 (79.9 - 98.4) | 2.8 |
| Sana’a | 68.4 | 74.0 (67.1 - 77.9) | 72.1 (63.3 - 78.5) | 93.1 (85.1 - 97.0) | 36.1 |
| Sana’a City | 57.4 | 54.7 (37.1 - 66.1) | 59.8 (38.9 - 76.4) | 50.9 (24.9 - 73.1) | -11.3 |
| Shabwah | 86.2 | 95.4 (90.7 - 97.9) | 87.6 (79.2 - 91.6) | 97.8 (94.1 - 99.2) | 13.5 |
| Taiz | 59.6 | 70.1 (59.3 - 80.3) | 70.4 (55.5 - 83.5) | 79.4 (58.6 - 92.5) | 33.2 |
| National | 72.0 | 83.0 (78.1 - 86.9) | 87.2 (80.2 - 92.2) | 93.8 (87.4 - 97.2) | 30.3 |

*2013 confidence intervals not provided by DHS.

Table S8: Under-5 mortality rate per 1,000 live births, 2013 - 2016, and percent change from 2013 to 2016, Yemen

| **Governorate** | **2013*** | **2014*** | **2015*** | **2016*** | **% change 2013 to 2016** |
| --- | --- | --- | --- | --- | --- |
| Abyan | 49 | 49 | 47.0 | 47.0 | -4.1 |
| Aden | 40 | 40 | 40.9 | 38.9 | -2.8 |
| Al-Baidha | 70 | 70 | 71.5 | 68.9 | -1.6 |
| Aldhalae | 47 | 47 | 46.1 | 45.1 | -4.0 |
| Al-Hodeida | 66 | 66 | 64.9 | 64.6 | -2.2 |
| Al-Jawf | 32 | 32 | 33.4 | 43.8 | 37.8 |
| Al-Mahrah | 52 | 52 | 50.4 | 49.5 | -5.0 |
| Al-Mahwit | 70 | 70 | 64.4 | 64.8 | -7.6 |
| Amran | 74 | 74 | 59.6 | 67.8 | -8.6 |
| Dhamar | 76 | 76 | 70.8 | 69.8 | -8.5 |
| Hadramout | 32 | 32 | 31.3 | 32.4 | 1.3 |
| Hajjah | 43 | 43 | 44.4 | 44.8 | 4.4 |
| Ibb | 62 | 62 | 59.4 | 58.4 | -6.0 |
| Lahj | 34 | 34 | 34.6 | 33.9 | -0.3 |
| Mareb | 61 | 61 | 95.3 | 88.8 | 47.7 |
| Reimah | 66 | 66 | 60.4 | 58.9 | -11.1 |
| Sadah | 54 | 54 | 109.0 | 88.9 | 67.7 |
| Sana’a | 71 | 71 | 72.0 | 81.3 | 15.1 |
| Sana’a City | 37 | 37 | 39.6 | 40.0 | 8.2 |
| Shabwah | 39 | 39 | 40.1 | 39.6 | 1.7 |
| Taiz | 60 | 60 | 66.0 | 67.2 | 12.4 |
| National | 53 | 53 | 56.4 | 56.8 | 7.3 |

*2013 confidence intervals not provided by DHS, 2014 – 2016 estimates were calculated based on additional deaths due to war and confidence intervals were not computed.

Table S9: Prevalence of underweight-BMI < 18.5 kg/m^2^ and 95% confidence intervals, 2013 - 2016, and percent change from 2013 to 2016 among women 15- 49 years old by governorate, Yemen

| **Governorate** | **2013 (95% CI)** | **2014 (95% CI)** | **2015 (95% CI)** | **2016 (95% CI)** | **% change 2013 to 2016** |
| --- | --- | --- | --- | --- | --- |
| Abyan | 21.3 (16.4 - 26.1) | 16.7 (13.7 - 19.2) | 18.9 (13.4 - 24.5) | 20.8 (14.0 – 31.1) | -2.4 |
| Aden | 15.7 (13.3 - 18.1) | 11.7 ( 8.3 - 16.5) | 14.9 ( 9.2 - 23.7) | 16.7 (9.8 – 27.3) | 6.2 |
| Al-Baidha | 10.9 (7.8 - 14.1) | 20.6 (16.3 - 24.1) | 23.1 (17.1 - 29.0) | 24.7 (17.9 – 30.9) | 126.7 |
| Aldhalae | 21.0 (17.8 - 24.1) | 19.2 (16.0 - 21.7) | 21.6 (17.6 - 24.8) | 23.1 (19.0 – 27.2) | 9.9 |
| Al-Hodeida | 40.3 (36.2 - 44.5) | 25.3 (19.8 - 29.9) | 26.9 (20.1 - 33.7) | 27.7 (19.1 – 38.0) | -31.2 |
| Al-Jawf | 20.3 (13.1 - 27.5) | 20.5 (16.3 - 29.0) | 22.3 (16.3 - 30.3) | 22.8 (17.9 – 31.1) | 12.1 |
| Al-Mahrah | 17.3 (11.7 - 22.9) | 14.1 ( 9.7 - 18.5) | 17.4 (14.8 - 19.5) | 18.0 (12.9 – 25.1) | 4.0 |
| Al-Mahwit | 32.1 (26.1 - 38.0) | 23.8 (20.4 - 27.7) | 25.1 (21.6 - 28.7) | 25.0 (21.6 – 29.1) | -22.0 |
| Amran | 25.0 (20.8 - 29.2) | 24.5 (20.1 - 29.5) | 26.5 (20.6 - 32.5) | 26.9 (22.8 – 30.5) | 7.4 |
| Dhamar | 23.2 (19.1 - 27.2) | 24.7 (20.3 - 29.7) | 26.4 (21.5 - 31.1) | 26.6 (22.2 – 30.6) | 14.8 |
| Hadramout | 20.1 (15.4 - 24.8) | 14.8 (10.2 - 18.9) | 17.9 (12.8 - 22.6) | 19.1 (12.9 – 24.9) | -5.2 |
| Hajjah | 40.0 (35.7 - 44.2) | 31.8 (24.8 - 36.9) | 31.9 (24.8 - 37.4) | 32.1 (24.1 – 39.7) | -19.7 |
| Ibb | 20.2 (16.6 - 23.7) | 20.2 (16.6 - 23.3) | 22.3 (18.2 - 26.1) | 22.8 (18.5 – 26.8) | 12.9 |
| Lahj | 21.4 (17.1 - 25.6) | 19.8 (14.3 - 27.6) | 21.6 (17.6 - 26.2) | 23.0 (15.9 – 33.0) | 7.3 |
| Mareb | 20.7 (16.6 - 24.9) | 19.9 (17.8 - 22.3) | 23.8 (17.6 - 32.3) | 24.9 (18.5 – 32.2) | 20.2 |
| Reimah | 25.5 (19.9 - 31.0) | 28.8 (24.3 - 32.1) | 29.3 (23.8 - 35.1) | 29.8 (21.4 – 40.1) | 16.7 |
| Sadah | 19.2 (15.6 - 22.8) | 26.9 (12.6 - 40.2) | 29.6 (18.8 - 37.2) | 31.7 (19.4 – 42.6) | 64.9 |
| Sana’a | 21.7 (18.5 - 25.0) | 23.1 (19.8 - 25.4) | 25.2 (21.3 - 28.2) | 27.2 (20.9 – 33.6) | 25.1 |
| Sana’a City | 15.7 (13.1 - 18.3) | 10.8 ( 6.6 - 14.5) | 13.8 ( 8.1 - 20.5) | 15.3 (8.1 – 24.6) | -2.8 |
| Shabwah | 20.6 (16.6 - 24.7) | 18.8 (11.0 - 24.0) | 21.9 (16.0 - 25.1) | 24.2 (14.9 – 34.9) | 17.5 |
| Taiz | 28.6 (24.9 - 32.3) | 18.8 (13.8 - 23.5) | 20.7 (14.2 - 27.6) | 23.9 (15.2 – 34.1) | -16.6 |
| National | 24.9 (23.8 - 26.0) | 20.8 (18.1 - 22.9) | 23.2 (19.1 - 27.3) | 24.6 (18.7 – 31.5) | -1.2 |

Table S10: Prevalence of moderate acute malnutrition – upper middle arm circumference 21 – 22.9 cm and 95% confidence intervals, 2013 - 2016, and percent change from 2013 to 2016 among women 15- 49 years old by governorate, Yemen

| **Governorate** | **2013*** | **2014 (95% CI)** | **2015 (95% CI)** | **2016 (95% CI)** | **% change 2013 to 2016** |
| --- | --- | --- | --- | --- | --- |
| Abyan | 14.8 | 17.1 (16.5 - 17.7) | 18.9 (18.5 - 19.3) | 20.4 (20.1 - 20.9) | 37.8 |
| Aden | 9.8 | 13.5 (12.6 - 14.2) | 15.6 (15.0 - 16.3) | 16.9 (16.4 - 17.6) | 72.2 |
| Al-Baidha | 11.9 | 19.9 (18.8 - 20.6) | 22.0 (20.9 - 23.0) | 23.5 (22.4 - 24.8) | 97.5 |
| Aldhalae | 18.6 | 19.1 (18.5 - 19.6) | 21.1 (20.6 - 21.5) | 22.4 (22.0 - 23.0) | 20.5 |
| Al-Hodeida | 21.1 | 24.3 (23.4 - 25.6) | 25.8 (24.9 - 27.4) | 26.6 (25.8 - 28.5) | 26.3 |
| Al-Jawf | 19.9 | 21.0 (20.0 - 22.0) | 22.5 (21.8 - 23.7) | 23.0 (22.3 - 24.2) | 15.5 |
| Al-Mahrah | 11.6 | 15.4 (14.3 - 15.9) | 17.8 (17.1 - 18.2) | 18.2 (17.7 - 18.7) | 57.1 |
| Al-Mahwit | 26.1 | 23.6 (23.2 - 24.1) | 24.9 (24.5 - 25.5) | 24.9 (24.5 - 25.5) | -4.8 |
| Amran | 28.1 | 24.3 (23.7 - 24.8) | 26.3 (25.8 - 27.0) | 26.7 (26.2 - 27.5) | -5.1 |
| Dhamar | 18.9 | 24.5 (24.0 - 25.0) | 26.2 (25.7 - 26.9) | 26.4 (25.9 - 27.2) | 39.9 |
| Hadramout | 15.1 | 15.5 (14.7 - 16.2) | 17.8 (17.1 - 18.4) | 18.7 (18.0 - 19.3) | 23.6 |
| Hajjah | 32.4 | 31.4 (30.5 - 34.0) | 31.5 (30.6 - 34.1) | 31.8 (30.8 - 34.4) | -2.0 |
| Ibb | 20.4 | 20.1 (19.6 - 20.5) | 21.9 (21.5 - 22.2) | 22.3 (22.0 - 22.7) | 9.5 |
| Lahj | 17.1 | 19.7 (19.3 - 20.1) | 21.2 (20.9 - 21.6) | 22.4 (22.1 - 22.9) | 31.2 |
| Mareb | 14.8 | 19.8 (19.4 - 20.2) | 23.2 (23.0 - 23.8) | 24.3 (24.0 - 25.0) | 64.1 |
| Reimah | 32.2 | 28.6 (28.2 - 30.0) | 29.2 (28.8 - 30.7) | 29.6 (29.2 - 31.2) | -8.1 |
| Sadah | 30.5 | 25.3 (23.3 - 27.5) | 28.1 (26.1 - 31.0) | 30.3 (28.3 - 33.8) | -0.7 |
| Sana’a | 21.9 | 22.4 (21.6 - 23.0) | 24.4 (23.7 - 25.4) | 26.3 (25.7 - 27.8) | 20.3 |
| Sana’a City | 14.4 | 13.1 (12.0 - 13.8) | 15.0 (14.2 - 15.7) | 16.0 (15.3 - 16.7) | 10.9 |
| Shabwah | 19.9 | 18.4 (17.2 - 19.3) | 21.0 (19.8 - 21.8) | 23.0 (21.9 - 24.3) | 15.7 |
| Taiz | 22.1 | 18.8 (18.3 - 19.2) | 20.3 (20.0 - 20.8) | 23.1 (22.7 - 23.8) | 4.4 |
| National | 20.4 | 20.4 (19.7 - 20.9) | 22.4 (21.8 - 23.1) | 23.7 (23.2 - 24.7) | 16.3 |

*2013 confidence intervals not provided by DHS.

Table S11: Prevalence of severe acute malnutrition – upper middle arm circumference <219 cm and 95% confidence intervals, 2013 - 2016, and percent change from 2013 to 2016 among women 15- 49 years old by governorate, Yemen

| **Governorate** | **2013*** | | **2014 (95% CI)** | **2015 (95% CI)** | **2016 (95% CI)** | **% change 2013 to 2016** |
| --- | --- | --- | --- | --- | --- | --- |
| Abyan | | 8.9 | 7.4 ( 5.6 - 8.7) | 7.9 ( 6.2 - 9.1) | 8.4 ( 6.7 - 9.5) | -5.5 |
| Aden | | 6.6 | 5.3 ( 3.1 - 6.9) | 5.9 ( 3.7 - 7.4) | 6.2 ( 3.9 - 7.9) | -6.0 |
| Al-Baidha | | 3.0 | 10.5 ( 7.5 - 13.6) | 11.3 ( 8.4 - 15.1) | 11.9 ( 8.8 - 16.1) | 296.0 |
| Aldhalae | | 11.3 | 12.6 (10.0 - 16.5) | 10.1 ( 8.6 - 11.5) | 10.6 ( 9.1 - 12.2) | -6.4 |
| Al-Hodeida | | 14.2 | 8.7 ( 5.0 - 12.1) | 13.2 (10.5 - 18.2) | 13.5 (10.9 - 19.3) | -4.8 |
| Al-Jawf | | 8.9 | 7.1 ( 4.7 - 8.7) | 9.2 ( 5.7 - 13.0) | 9.3 ( 5.8 - 13.2) | 4.9 |
| Al-Mahrah | | 5.4 | 11.5 ( 8.1 - 14.3) | 7.8 ( 6.1 - 9.1) | 8.0 ( 6.3 - 9.2) | 47.8 |
| Al-Mahwit | | 11.6 | 9.4 ( 7.7 - 11.1) | 12.0 ( 8.7 - 14.8) | 11.9 ( 8.7 - 14.8) | 3.0 |
| Amran | | 20.4 | 13.1 ( 8.8 - 16.7) | 13.9 ( 9.8 - 17.9) | 14.0 (10.0 - 18.2) | -31.3 |
| Dhamar | | 10.0 | 13.3 ( 8.9 - 17.1) | 14.0 ( 9.7 - 18.1) | 14.1 ( 9.9 - 18.3) | 40.6 |
| Hadramout | | 11.0 | 7.1 ( 5.0 - 9.1) | 7.9 ( 5.7 - 9.7) | 8.2 ( 5.9 - 10.0) | -25.8 |
| Hajjah | | 23.8 | 17.8 (13.8 - 27.7) | 17.9 (13.9 - 27.8) | 18.0 (13.9 - 28.3) | -24.4 |
| Ibb | | 8.2 | 9.7 ( 7.9 - 11.1) | 10.3 ( 8.8 - 11.7) | 10.5 ( 9.0 - 11.8) | 28.1 |
| Lahj | | 8.8 | 8.9 ( 7.3 - 10.1) | 9.4 ( 8.0 - 10.5) | 9.8 ( 8.3 - 11.0) | 11.3 |
| Mareb | | 5.1 | 9.1 ( 7.4 - 10.4) | 10.3 ( 8.8 - 11.8) | 10.6 ( 9.2 - 12.6) | 108.3 |
| Reimah | | 15.6 | 14.7 (11.3 - 19.4) | 15.0 (11.4 - 20.2) | 15.1 (11.6 - 20.8) | -2.9 |
| Sadah | | 13.8 | 15.5 ( 9.6 - 25.5) | 16.8 (10.7 - 28.6) | 17.8 (11.6 - 32.0) | 28.7 |
| Sana’a | | 10.6 | 12.0 ( 9.5 - 14.3) | 12.8 (10.5 - 16.0) | 13.5 (11.2 - 17.9) | 27.7 |
| Sana’a City | | 6.8 | 5.0 ( 2.9 - 6.5) | 5.5 ( 3.4 - 6.9) | 5.8 ( 3.7 - 7.1) | -14.8 |
| Shabwah | | 15.5 | 10.2 ( 7.2 - 13.9) | 11.3 ( 8.1 - 14.9) | 12.1 ( 8.9 - 16.3) | -22.1 |
| Taiz | | 13.2 | 8.1 ( 6.4 - 9.3) | 8.6 ( 6.8 - 9.7) | 9.4 ( 7.5 - 11.3) | -28.6 |
| National | | 11.6 | 10.2 ( 8.3 - 11.9) | 10.9 ( 9.1 - 12.8) | 11.4 ( 9.7 - 14.0) | -1.6 |

*2013 confidence intervals not provided by DHS.

Table S12: Maternal mortality rate per 100,000 live births, 2013 - 2016, and percent change from 2013 to 2016, Yemen

| **Governorate** | **2013*** | **2014*** | **2015*** | **2016*** | **% change 2013 to 2016** |
| --- | --- | --- | --- | --- | --- |
| Abyan | 162.6 | 161.4 | 164.0 | 164.5 | 1.2 |
| Aden | 79.9 | 79.3 | 80.5 | 80.8 | 1.2 |
| Al-Baidha | 196.1 | 194.6 | 197.9 | 198.6 | 1.3 |
| Aldhalae | 201.8 | 200.3 | 203.5 | 204.2 | 1.2 |
| Al-Hodeida | 240.3 | 238.5 | 242.3 | 243.1 | 1.2 |
| Al-Jawf | 266.2 | 264.2 | 268.5 | 270.0 | 1.4 |
| Al-Mahrah | 143.5 | 142.5 | 144.7 | 144.7 | 0.8 |
| Al-Mahwit | 273.4 | 271.4 | 275.6 | 275.8 | 0.9 |
| Amran | 301.6 | 299.3 | 304.1 | 305.2 | 1.2 |
| Dhamar | 310.1 | 307.8 | 312.6 | 313.2 | 1.0 |
| Hadramout | 126.0 | 125.0 | 127.0 | 127.3 | 1.1 |
| Hajjah | 319.5 | 317.1 | 322.5 | 323.9 | 1.4 |
| Ibb | 213.6 | 211.9 | 215.3 | 215.7 | 1.0 |
| Lahj | 200.6 | 199.1 | 202.4 | 203.1 | 1.2 |
| Mareb | 239.7 | 237.9 | 245.6 | 248.2 | 3.5 |
| Reimah | 309.0 | 306.6 | 311.5 | 312.2 | 1.0 |
| Sadah | 264.2 | 262.2 | 269.4 | 271.9 | 2.9 |
| Sana’a | 248.4 | 246.5 | 251.0 | 252.5 | 1.7 |
| Sana’a City | 78.9 | 78.3 | 79.6 | 79.9 | 1.3 |
| Shabwah | 189.4 | 188.0 | 191.1 | 191.8 | 1.3 |
| Taiz | 225.1 | 223.4 | 227.1 | 230.6 | 2.4 |
| National | 210.6 | 209.0 | 212.5 | 213.4 | 1.3 |

*2013 – 2016 estimates were calculated directly based on GBD maternal mortality estimates and change in estimates. Confidence intervals were not computed.
