# Supplementary material for: Health in Yemen: losing ground in war time
Source: Global Health. 2018 Apr 25;14:42. doi: 10.1186/s12992-018-0354-9 (PMC5918919; doi:10.1186/s12992-018-0354-9)
Supplement: Supplementary file 5 — [A] Maps of maternal and child health indicators' estimates in 2016; [B] percent change from 2013 and 2016; [C] estimates in 2013, 2014, 2015, and 2016. In panel [C], the boxes indicate the 25th, 50th, and 75th percentile across all governorates, while the lines indicate the full range across governorates and the dots indicate national-level estimates. (DOCX 1389 kb) [file 12992_2018_354_MOESM5_ESM.docx]

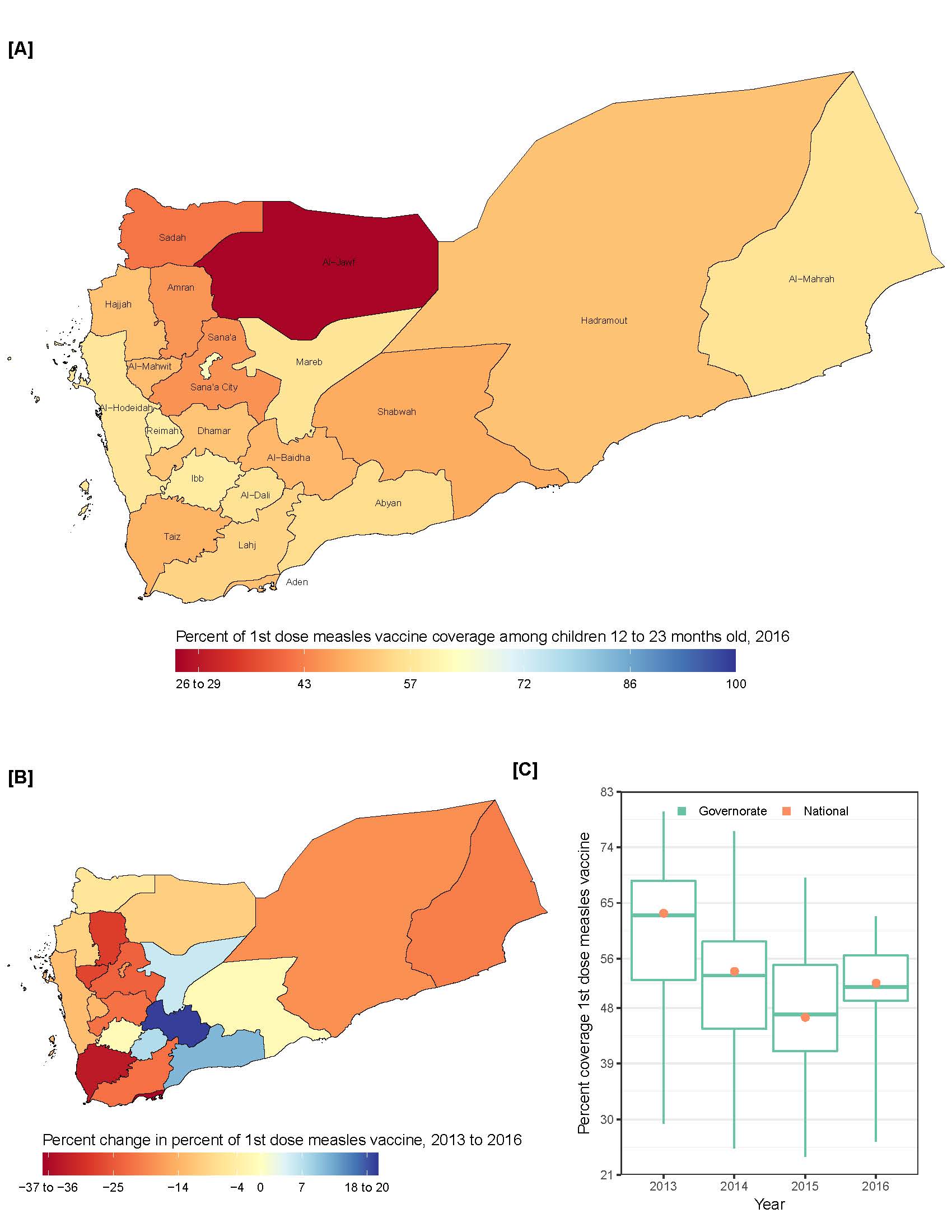


Figure S1: [A] First-dose measles vaccine coverage in 2016; [B] percent change in frst-dose measles vaccine coverage from 2013 to 2016; [C] first-dose measles vaccine coverage in 2013, 2014, 2015, and 2016. In panel [C], the boxes indicate the 25th, 50th, and 75th percentile across all governorates while the lines indicate the full range across governorates and the dots indicate the national-level coverage


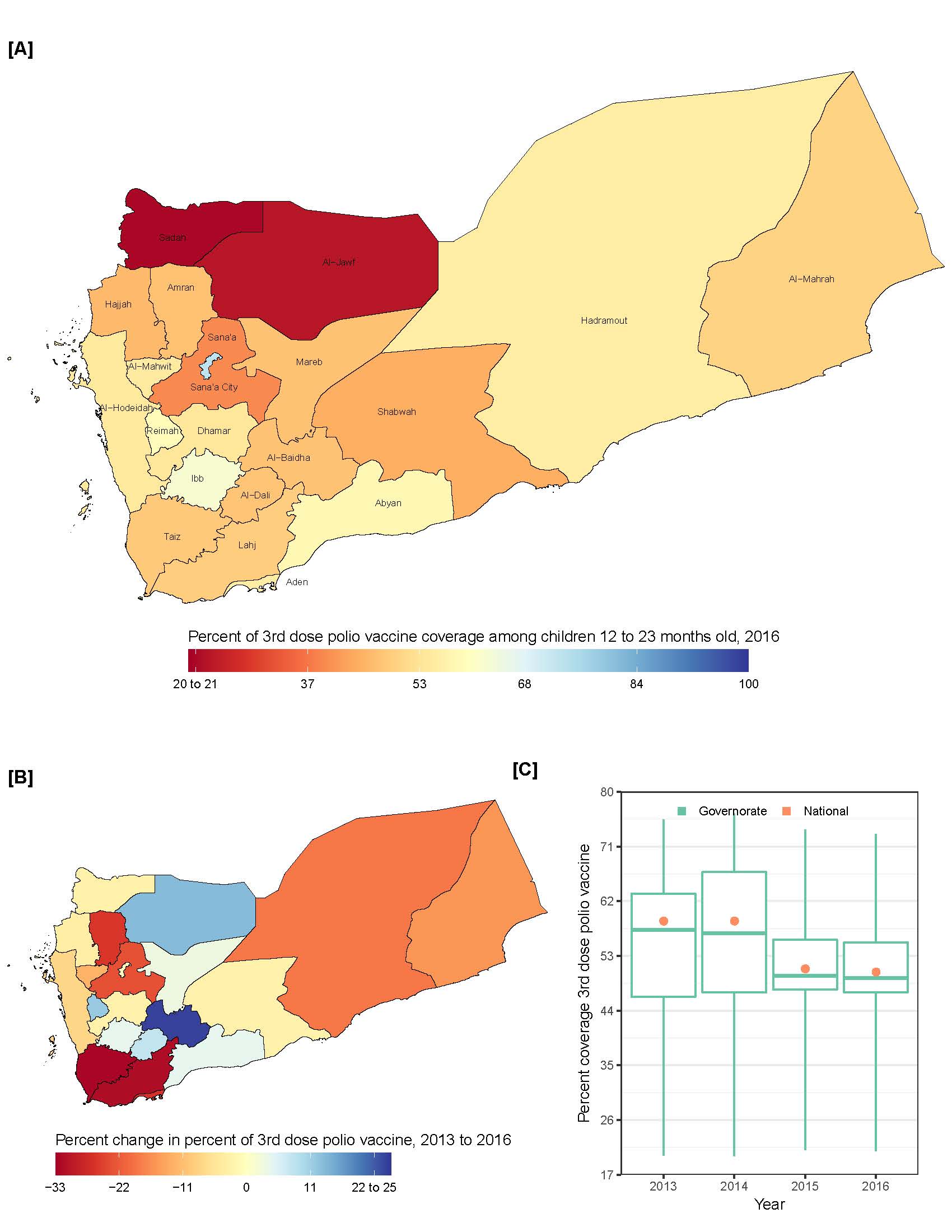


Figure S2: [A] Third-dose polio vaccine coverage in 2016; [B] percent change in third-dose polio vaccine coverage from 2013 to 2016; [C] third-dose polio vaccine coverage in 2013, 2014, 2015, and 2016. In panel [C], the boxes indicate the 25th, 50th, and 75th percentile across all governorates while the lines indicate the full range across governorates and the dots indicate the national-level coverage


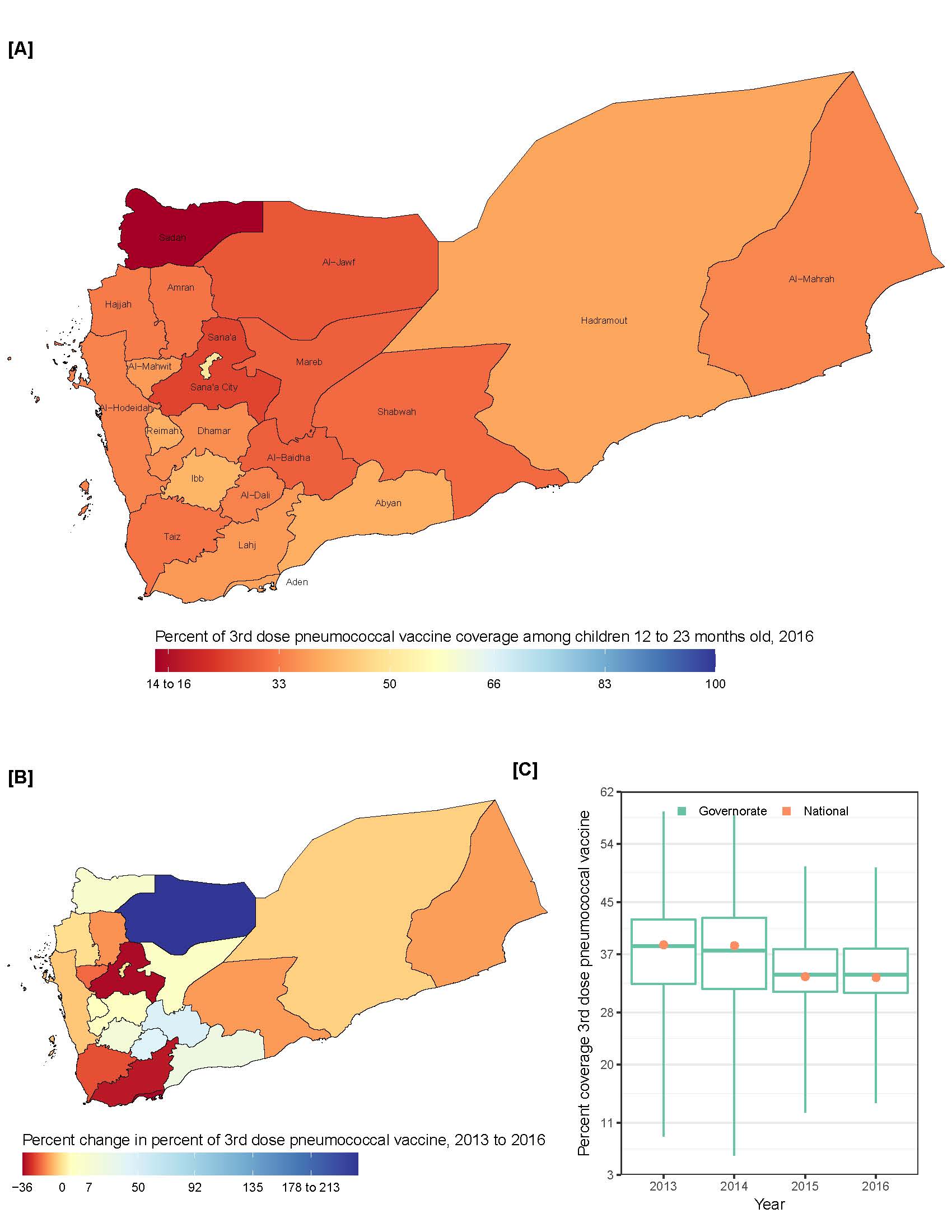


Figure S3: [A] Third-dose pneumococcal vaccine coverage in 2016; [B] percent change in third-dose pneumococcal vaccine coverage from 2013 to 2016; [C] third-dose pneumococcal vaccine coverage in 2013, 2014, 2015, and 2016. In panel [C], the boxes indicate the 25th, 50th, and 75th percentile across all governorates while the lines indicate the full range across governorates and the dots indicate the national-level coverage


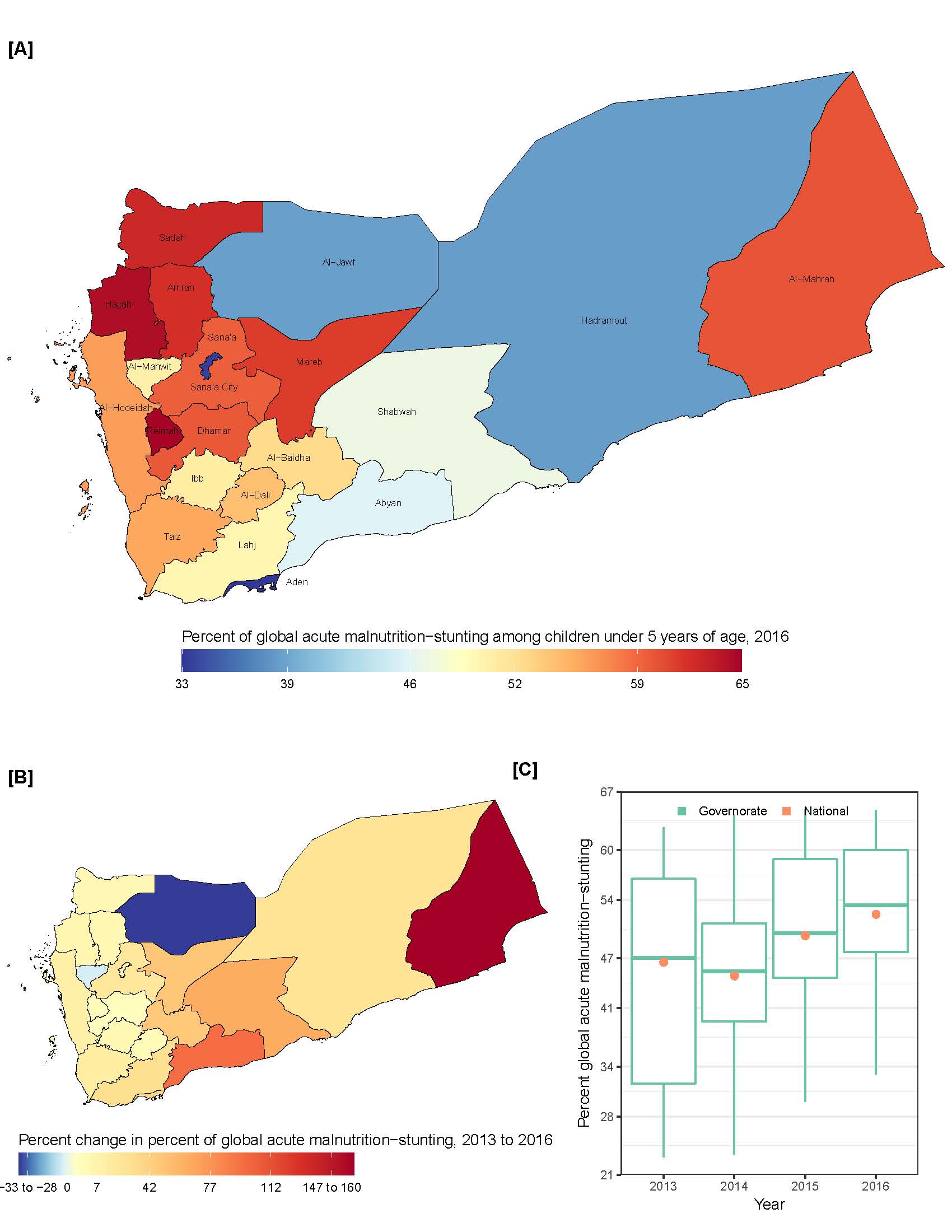


Figure S4: [A] Prevalence of global acute malnutrition – stunting in 2016; [B] percent change in Prevalence of global acute malnutrition – stunting from 2013 to 2016; [C] Prevalence of global acute malnutrition – stunting in 2013, 2014, 2015, and 2016. In panel [C], the boxes indicate the 25th, 50th, and 75th percentile across all governorates while the lines indicate the full range across governorates and the dots indicate the national-level coverage


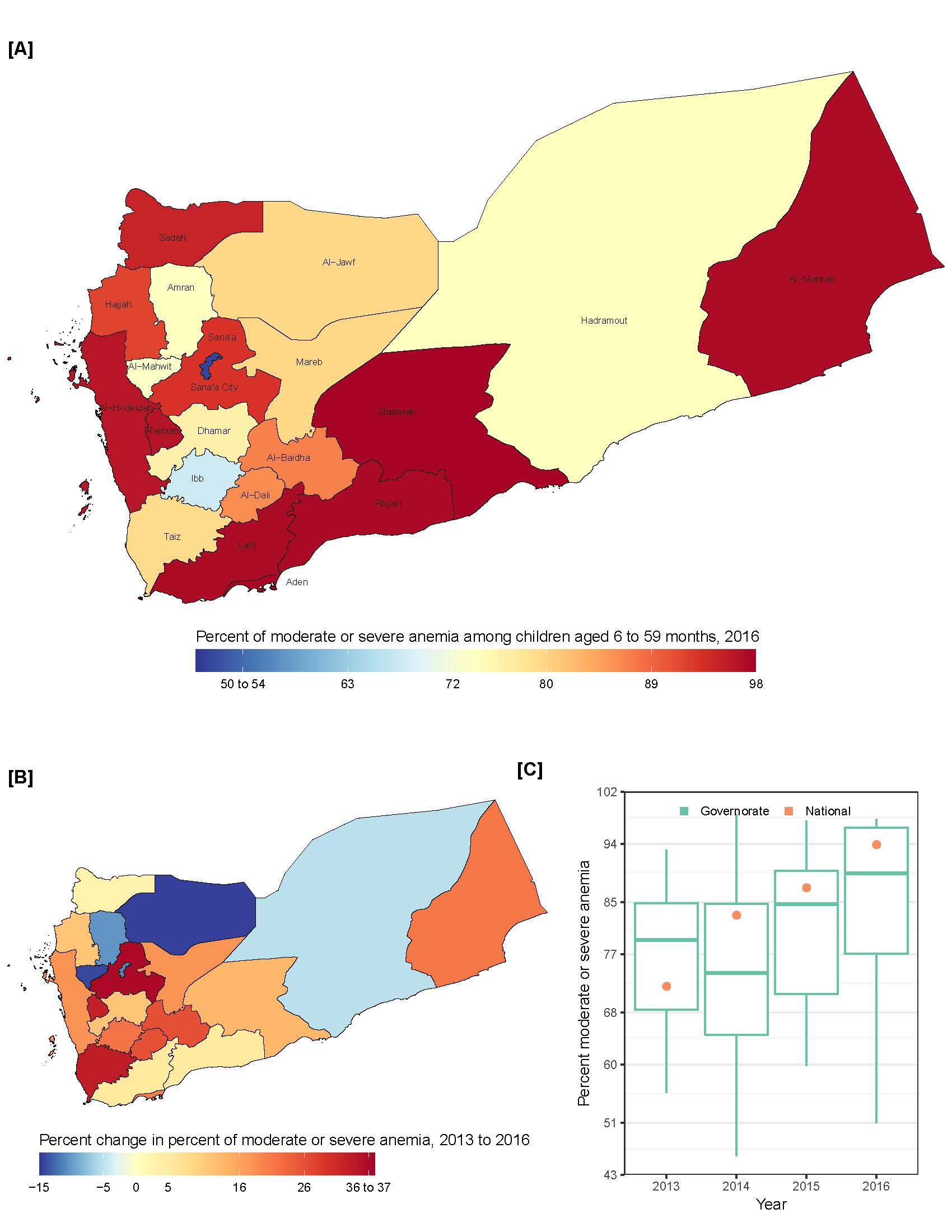


Figure S5: [A] Prevalence of child anemia in 2016; [B] percent change in child anemia from 2013 to 2016; [C] Prevalence of child anemia in 2013, 2014, 2015, and 2016. In panel [C], the boxes indicate the 25th, 50th, and 75th percentile across all governorates while the lines indicate the full range across governorates and the dots indicate the national-level coverage


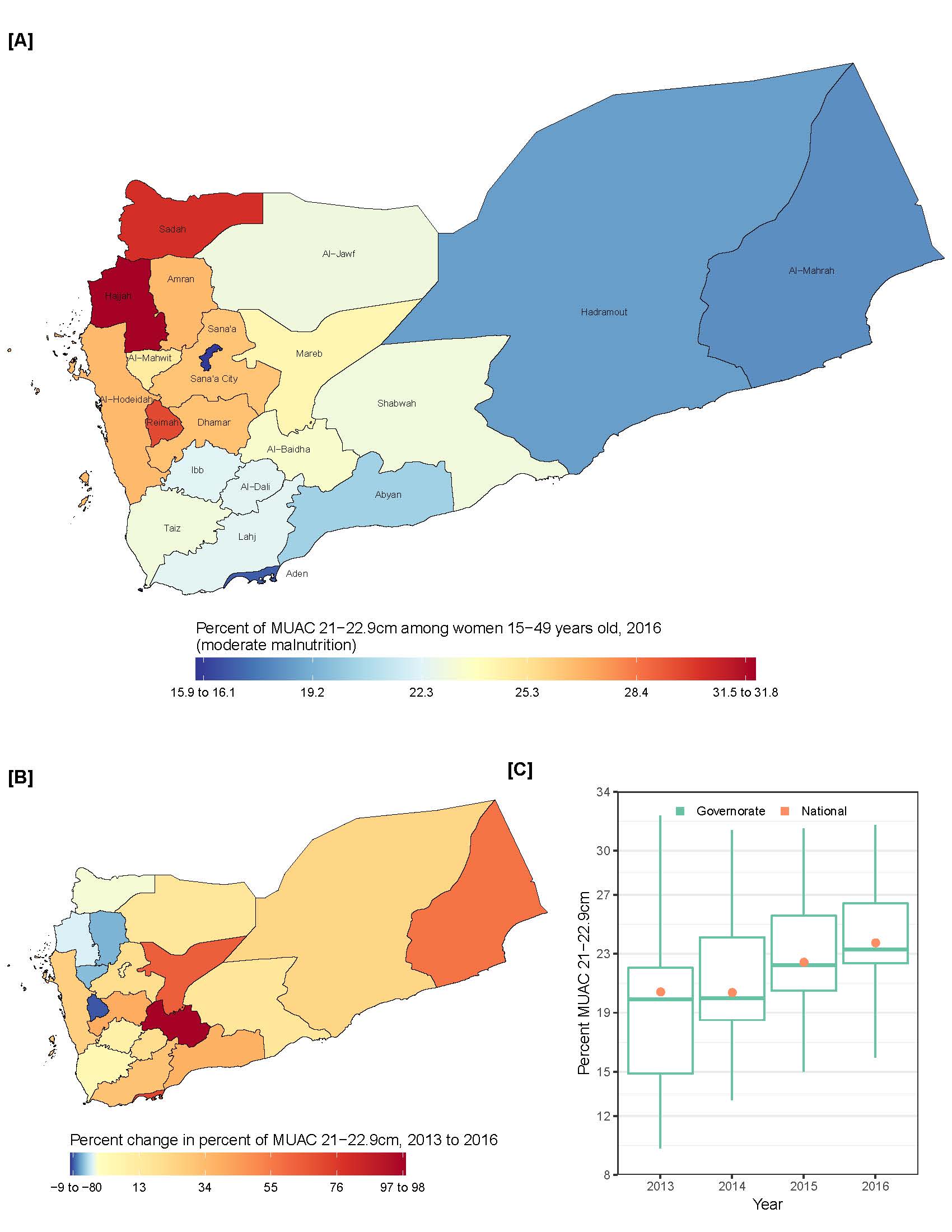


Figure S6: [A] Prevalence of maternal moderate malnutrition – middle upper arm circumference 21 – 22.9 cm in 2016; [B] percent change in maternal moderate malnutrition from 2013 to 2016; [C] Prevalence of maternal moderate malnutrition in 2013, 2014, 2015, and 2016. In panel [C], the boxes indicate the 25th, 50th, and 75th percentile across all governorates, while the lines indicate the full range across governorates and the dots indicate national-level coverage.


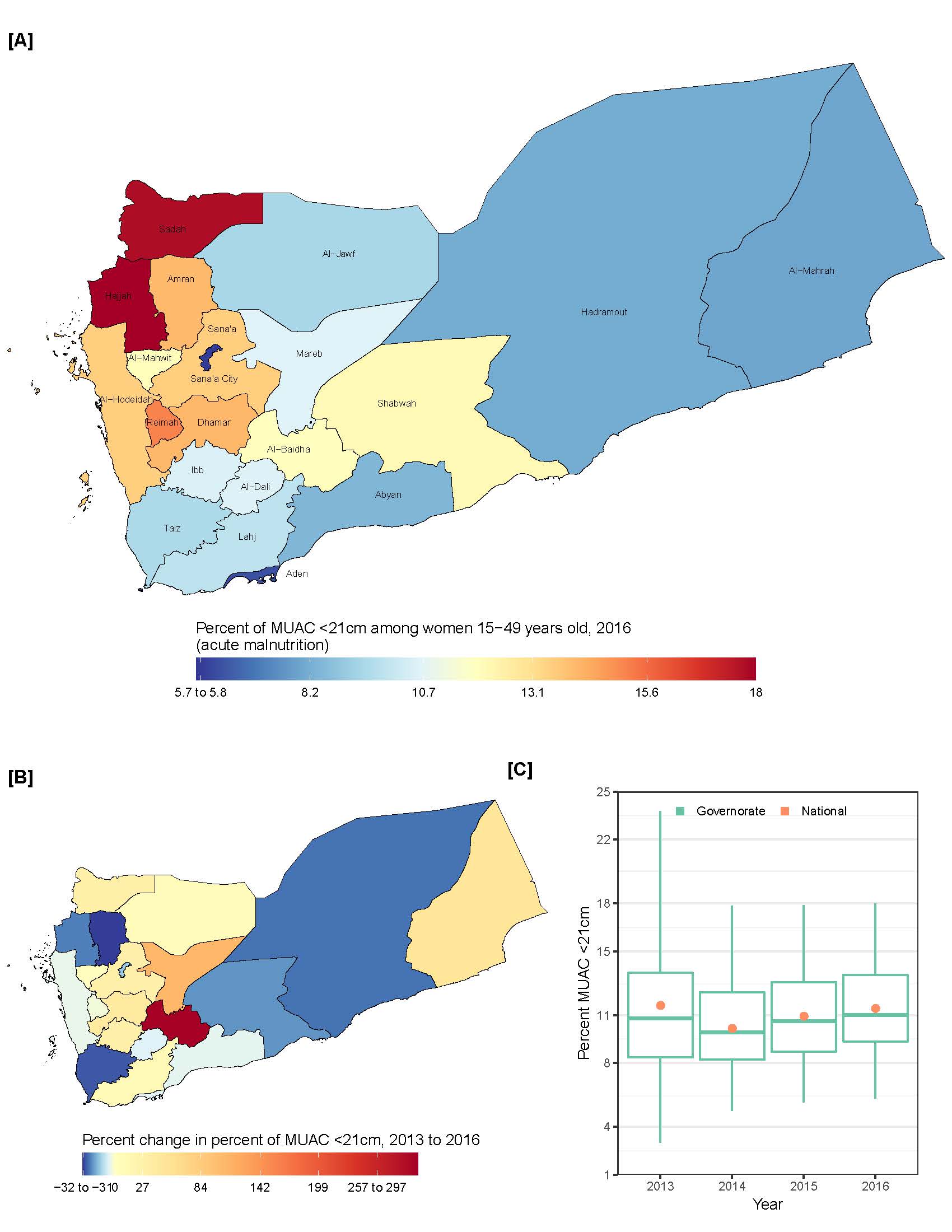


Figure S7: [A] Prevalence of maternal severe malnutrition – middle upper arm circumference < 21 cm in 2016; [B] percent change in maternal severe malnutrition from 2013 to 2016; [C] Prevalence of maternal severe malnutrition in 2013, 2014, 2015, and 2016. In panel [C], the boxes indicate the 25th, 50th, and 75th percentile across all governorates, while the lines indicate the full range across governorates and the dots indicate national-level coverage.
